# KDM6B is an androgen regulated gene and plays oncogenic roles by demethylating H3K27me3 at cyclin D1 promoter in prostate cancer

**DOI:** 10.1038/s41419-020-03354-4

**Published:** 2021-01-06

**Authors:** Zhi Cao, Xiaolei Shi, Feng Tian, Yu Fang, Jason Boyang Wu, Stefan Mrdenovic, Xinwen Nian, Jin Ji, Huan Xu, Chen Kong, Yalong Xu, Xi Chen, Yuhua Huang, Xuedong Wei, Yongwei Yu, Bo Yang, Leland W. K. Chung, Fubo Wang

**Affiliations:** 1 Department of Urology, Changhai Hospital, Navy Medical University, 168 Changhai Road, Shanghai, 200433 China; 2 Department of Urology, The Eighth People’s Hospital of Shanghai, 8 Caobao Road, Shanghai, 200235 China; 3 Department of Pharmaceutical Sciences, College of Pharmacy and Pharmaceutical Sciences, Washington State University, Spokane, WA 99202 USA; 4 Uro-Oncology Research Program, Samuel Oschin Comprehensive Cancer Institute, Department of Medicine, Cedars-Sinai Medical Center, Los Angeles, CA 90048 USA; 5 Division of Hematology, Department of Internal Medicine, University Hospital Osijek, Osijek, Croatia; 6 Department of Traditional Chinese Medicine, New Jiangwan City Community Health Service Centre, Shanghai, China; 7 Department of Urology, The First Affiliated Hospital of Soochow University, 188 Shizi Road, Suzhou, 215006 Jiangsu China; 8 Department of Pathology, Changhai Hospital, Navy Medical University, 168 Changhai Road, Shanghai, 200433 China

**Keywords:** Prostate cancer, Mechanisms of disease, Target identification, DNA methylation

## Abstract

Lysine (K)-specific demethylase 6B (KDM6B), a stress-inducible H3K27me3 demethylase, plays oncogenic or antitumoral roles in malignant tumors depending on the type of tumor cell. However, how this histone modifier affects the progression of prostate cancer (PCa) is still unknown. Here we analyzed sequenced gene expression data and tissue microarray to explore the expression features and prognostic value of KDM6B in PCa. Further, we performed in vitro cell biological experiments and in vivo nude mouse models to reveal the biological function, upstream and downstream regulation mechanism of KDM6B. In addition, we investigated the effects of a KDM6B inhibitor, GSK-J4, on PCa cells. We showed that KDM6B overexpression was observed in PCa, and elevated KDM6B expression was associated with high Gleason Score, low serum prostate-specific antigen level and shorted recurrence-free survival. Moreover, KDM6B prompted proliferation, migration, invasion and cell cycle progression and suppressed apoptosis in PCa cells. GSK-J4 administration could significantly suppress the biological function of KDM6B in PCa cells. KDM6B is involved in the development of castration-resistant prostate cancer (CRPC), and combination of MDV3100 plus GSK-J4 is effective for CRPC and MDV3100-resistant CRPC. Mechanism exploration revealed that androgen receptor can decrease the transcription of KDM6B and that KDM6B demethylates H3K27me3 at the cyclin D1 promoter and cooperates with smad2/3 to prompt the expression of cyclin D1. In conclusion, our study demonstrates that KDM6B is an androgen receptor regulated gene and plays oncogenic roles by promoting cyclin D1 transcription in PCa and GSK-J4 has the potential to be a promising agent for the treatment of PCa.

## Introduction

Histone methylation can change the steric configuration of DNA, leading to gene expression or repression^1^. Histone lysine residues can be mono-, di-, or trimethylated, and the different degrees of methylation on a particular site could be linked to different functional outcomes^2^. H3K27 trimethylation (H3K27me3) is one of the most important repressive histone modifications and is mediated by the histone methyltransferase enhancer of zeste homolog 2 (EZH2)^3^. EZH2 is the catalytic subunit of the polycomb repressive complex (PRC2) and H3K27me3 contributes to the recruitment of the PRC1 complex. PRC1 and PRC2 repress the expression of various developmental genes^4^.

Lysine (K)-specific demethylase 6B (KDM6B), also called jumonji domain-containing protein D3 (JMJD3), could counter the effect of EZH2. It is well known that KDM6B, a member of the Jumonji C (JmjC) histone demethylase family, contains a JmjC domain and can remove all three mono-, di-, or trimethyl groups from methylated H3K27^5,6^. It is involved in multiple cellular processes, including differentiation, proliferation, senescence, and apoptosis, which tend to effect responses, such as vertebrate development, cancer, inflammatory diseases, and neurodegenerative diseases^5–9^. *KDM6B* expression could be induced by certain normal developmental cues and by stressful or pathogenic factors, such as inflammatory cytokines, cancerogenic factors, and mitochondrial stress inducers^10,11^. This is different from ubiquitously transcribed X chromosome tetratricopeptide repeat protein (also named KDM6A), which is a constitutively expressed H3K27 demethylase in various tissue types^12,13^.

The vital role of *KDM6B* in the development of malignant hematopoiesis has been widely detailed. Previous studies have reported the association between the abnormal elevation of KDM6B and the overactivated nuclear factor-κb (NF-κb)/innate immunity pathway in human CD34+ hematopoietic stem cells of myelodysplastic syndrome^14^, the oncogenic role of KDM6B in regulating immune cell differentiation and the immunological responses in lymphoid malignancies^15–17^, and the oncogenic activity of KDM6B in NOTCH1-driven human T-cell acute lymphocytic leukemia (T-ALL)^18^. Moreover, the abnormal expression or activity of KDM6B was observed in solid tumors, including cervical carcinoma^19^, pancreatic carcinoma^20,21^, glioma^22,23^, and renal cancer^24^. *KDM6B* expression is also upregulated in prostate cancer (PCa) and is further increased during metastasis^25^. Moreover, it has also been reported that global histone modification patterns can predict the risk of PCa occurrence and recurrence^26,27^, which indicates the great importance of histone modifications for the generation and development of PCa. However, the detailed effect and mechanism of KDM6B in PCa are still unclear.

Thus, we designed this study to observe alterations in *KDM6B* expression and its effect on PCa. The up- and downstream signaling pathways are also explored in this report. Further, the effects of a KDM6B inhibitor, GSK-J4, on PCa cells were also investigated. Considering the complexity of the internal environment in organisms, a subcutaneous PCa nude mouse model was applied to reveal the effects of KDM6B on PCa progression in vivo.

## Materials and methods

### Database analysis

The PCa gene expression data in The Cancer Genome Atlas (TCGA) database were manually retrieved from the UCSC Cancer Genomics Browser (https://genome-cancer.ucsc.edu) and five sets of data were downloaded from the GEO database (GSE6919, GSE35998, GSE6752, GSE46602, and GSE62872; https://www.ncbi.nlm.nih.gov/geo/). The Universal Expression Code approach was used to calculate the normalized expression as previously reported^28^. RNA sequencing of our Dingtianlidi program was conducted by Novogene Biotechnology (Beijing, China), and the detailed sequencing procedures and data analysis methods are presented in the Supplemental Materials.

### Tissue microarray construction and patients’ follow-up

This project was approved by the Clinical Research Ethics Committee of Shanghai Changhai Hospital of Naval Medical University. All of the clinical samples were obtained from Shanghai Changhai Hospital (Shanghai, China). Written informed consents were obtained from the participants before sampling. Construction of the tissue microarray was based on PCa and benign prostate hyperplasia histological specimens retrieved from the archives of the Department of Pathology at Changhai Hospital. Our pathology team identified the most representative area of each sample, and both the tumor tissue and the para-cancerous tissue were identified for the prostatectomy samples. The patients’ clinical and pathological data were obtained from the medical records. Different methods were applied to obtain the follow-up information as follows: reviewing the outpatient electronic medical record system and the PCa database of our department, phoning the patients or their family members, and sending letters or emails. A sustained elevation of the serum total prostate-specific antigen (PSA) level above 0.2 ng ml^−1^ on two or more occasions was defined as biochemical recurrence (BCR) and the date of BCR was assigned as the first date of total PSA levels >0.2 ng ml^−1^.

### Cell culture, transfection, and CCK-8 analysis

The PC3 (RRID: CVCL_0035), C42B (RRID: CVCL_4784), and LNCaP (RRID: CVCL_0395) cell lines were bought from American Tissue Culture Collection and stored in the cell lines bank of our laboratory. Materials of cell authentication was uploaded as Supplemental Data. The human PCa cell line PC3 was maintained with F12 medium (GIBCO, 21127-022) supplemented with 10% fetal bovine serum, and the LNCaP cell line and C42B cell line was cultured with 1640 medium (GIBCO, 11875-093) supplemented with 10% fetal bovine serum. The small interfering RNA (siRNA) transfection was facilitated by Lipofectamine RNAiMAX reagent (Thermo Fisher Scientific, 13778-150) according to the manufacturer’s instructions. The details of the siRNA sequence are presented in the Supplemental Materials. LNCaP cells were transfected with either pSLenti-EF1-Puro-CMV-*KDM6B*-3xFLAG or the control vector to construct *KDM6B* overexpression or negative control cell lines. The cells were seeded into 96-well plates and cell proliferation was detected using a CCK-8 kit (Shanghai Dojindo Company, CK-04) according to the manufacturer’s protocol.

### Migration and invasion assay

After siRNA transfection for 24 h, 5–8 × 10^4^ cells were seeded on a transwell insert (Corning, USA) in 100 μl culture medium (without fetal bovine serum) and were incubated for 1–2 days at 37 °C. The cells that adhered to the lower surface of the transwell insert were fixed with methanol, stained with a crystal violet solution, and counted under a microscope.

### Animal experiments

All animal experiments were approved by the Laboratory Animal Ethics Committee of the Second Military Medical University with the Guidelines for Animal Health and Use (Ministry of Science and Technology, China, 2006) and performed inside a biosafety cabinet during the animal’s light time cycle on the fourth floor of the Experimental Animal Room at Navy Medical University. Four-week-old male Nu/nu athymic nude mice (RRID: MGI:5649767) were bought from Sippr-BK laboratory animal Co. Ltd (Shanghai, China) and raised under specific pathogen free (SPF) room conditions for feeding and observation. A total of 5 × 10^6^ PCa cells per mouse were applied to construct the subcutaneous models in 36 mice. No blind method was applied in the research and random number method was applied to randomly divide all mice into the following six groups (*n* = 6 mice per group): (1) PC3 cell line + negative control siRNA, (2) PC3 cell line + *KDM6B* siRNA, (3) C42B cell line + negative control siRNA, (4) C42B cell line + *KDM6B* siRNA, (5) C42B cell line + dimethylsulfoxide (DMSO) (negative control), and (6) C42B cell line + GSK-J4 (50 mg/kg). For group 2 and group 4, the in vivo-jetPEI Delivery Kit (Polyplus, 201-10 G) and the *KDM6B* siRNA were used to knock down the expression of *KDM6B* by an intratumor injection according to the manufacturer’s protocol. Intratumor injection of in vivo-jetPEI Delivery Kit and negative control siRNA was conducted in group 1 and group 3. GSK-J4 was administrated through intraperitoneal injection with the concentration of 50 mg/kg and DMSO was taken as the control. The siRNA and drug delivery were conducted every 3 days. Tumor volume was measured using a Vernier calliper every 3 days and the tumor volume was calculated with the formula: 0.5 × length × width^2^. Nasal anesthesia with isoflurane was introduced to alleviate pain to the mice throughout experimental studies. At the end of the animal experiments, subcutaneous tumors were collected from the killed mice for further analysis.

### RNA sequencing and data processing

A total amount of 3 µg RNA of C42B or C42B *KDM6B* KD cell line was used as input material for the RNA sample preparations. Sequencing libraries were generated using NEBNex^®^ UltraTMRNA Library Prep Kit for Illumina^®^ (NEB, USA), following the manufacturer’s recommendations, and index codes were added to attribute sequences to each sample. Clustering of the indexed samples was performed on a cBot Cluster Generation System using a HiSeq X PE Cluster Kit V2.5 (Illumina) according to the manufacturer’s instructions. After cluster generation, the library preparations were sequenced on an Illumina Hiseq platform and 125 bp/150 bp paired-end reads were generated. We then selected Hisat2 as the mapping tool for which Hisat2 can generate a database of splice junctions based on the gene model annotation file and thus a better mapping result than other non-splice mapping tools. Differential expression analysis between C42B and C42B *KDM6B* KD (three biological replicates per condition) was performed using the DESeq2 R package (1.16.1). Genes with an adjusted *P*-value < 0.05 found by DESeq2 were assigned as differentially expressed. RNA-sequencing data were deposited into CNGB Sequence Archive (https://db.cngb.org/cnsa/) of CNGBdb with accession number CNP0001447.

### Flow cytometry assay

The cells were collected 48 h after siRNA transfection or drug treatment. The samples for the cell cycle analysis were stained with propidium iodide and the samples for the apoptosis analysis were stained with Annexin V-fluorescein isothiocyanate and propidium iodide. Then, the samples were analyzed using the MACS Quant Analyzer 10 flow cytometer (Miltenyi Biotec, Bergisch Gladbach, Germany). The data were analyzed using FlowJo software (Tree Star, Inc., San Carlos, USA).

### RNA isolation and quantitative real-time PCR

The total RNA was extracted using the RNeasy Mini Kit (Qiagen, 74106) and reverse-transcription PCR (RT-PCR) was performed with the ReverTra Ace qPCR RT Kit (Toyobo, FSQ-101) according to the manufacturer’s instructions. A real-time PCR assay was then conducted with SYBR green dye (Toyobo, QPK-201) on a StepOne Sequence Detection System (Applied Biosystems, Waltham, MA, USA). The 2^−ΔΔCT^ formula was then utilized to calculate the relative expression of the genes and β-actin was used as an internal control. The primers information is listed in the Supplemental Materials.

### Western blotting

The total protein content was isolated with an appropriate volume of a radio immunoprecipitation assay (RIPA) lysis buffer and protease inhibitor cocktail, which was then mixed with loading buffer and heated at 100 °C. Western blotting was performed according to the following classic procedures: electrophoresis on SDS-polyacrylamide gel electrophoresis, transfer onto polyvinylidene difluoride membranes (Millipore, IPVH00010), antibody incubation, and color development. The antibodies used in this study are as follows: anti-KDM6B (Abcam, ab154126), anti-β-actin (Proteintech, 60008-1), anti-cyclin D1 (CCND1) (Abgent, AP2612d), and anti-smad2/3 (Santa Cruz Biotechnology, sc-398844).

### Immunohistochemistry

Immunohistochemistry was conducted to assess the protein expression of KDM6B (Abcam, ab38113), ARs (Cell Signaling Technology, 5153), and CCND1 (Abgent, AP2612d). The expression levels of KDM6B, ARs, and CCND1 were quantified using the Pannoramic Viewer and Quant Center image analysis software (3D HISTECH, Budapest, Hungary) to calculate the H-score. The H-score was calculated as follows: percent of weak staining (scale: 0–100) × 1 + percent of moderate staining (scale: 0–100) × 2 + percent of strong staining (scale: 0–100) × 3.0. Then, the H-score was applied to assist pathologists in categorizing the KDM6B expression into four levels: negative, low, moderate, and high.

### ChIP assay

Chromatin immunoprecipitation (ChIP) assays were conducted by the Simple ChIP Plus Enzymatic Chromatin IP Kit (Cell Signaling Technology, 91820) according to the manufacturer’s instructions. Chromatin was immunoprecipitated using anti-AR (Cell Signaling Technology, 5153), anti-KDM6B (Abcam, ab38113), and anti-H3K27me3 (Abcam, ab6002) antibodies. An anti-histone H3 antibody (Cell Signaling Technology, 4620) and a normal rabbit IgG antibody (Cell Signaling Technology, 2729) were used as the positive control and the negative control, respectively. The ChIP-derived DNA was quantified using quantitative RT-PCR and the related primers are listed in the Supplemental Materials.

### Dual-luciferase reporter assay

293T cells were first transfected with *androgen receptor* (*AR*) plasmid and its control plasmid. Then, these cells were cotransfected with pGiro dual-luciferase reporter and pGL4.10-*KDM6B* promoter (wild type (WT) or mutation (MUT)) with Lipofectamine 2000 (Invitrogen). Six times were repeated in each group. The luciferase activity was detected by Dual-Luciferase Reporter Assay System (Promega) after 48 h of transfection. *Renilla* luciferase activity was normalized against firefly luciferase activity.

### Immunoprecipitation

A protein lysate (1 mg) was incubated for 2 h with protein G-conjugated magnetic beads (Dynabeads, Santa Cruz Biotechnology, sc-2003) to reduce nonspecific binding and was then centrifuged to remove the protein G-conjugated magnetic beads. The supernatant was incubated with the primary antibody overnight followed by a 2 incubation with the protein G-conjugated magnetic beads. And then the compounds of protein and magnetic beads were centrifuged and collected. The immunoprecipitated proteins were assessed by western blotting.

### Mass spectrometric analysis

The mass spectrometric analysis was conducted at AIMS Scientific Co., Ltd (Shanghai, China). First, the total protein was isolated from the PC3 cell line and KDM6B-targeted immunoprecipitation was conducted. Then, the peptide samples were analyzed with a Thermo Fisher LTQ Obitrap ETD mass spectrometer. The details of the mass spectrometry and bioinformatic analyses are presented in the Supplemental Materials.

### Statistical analysis

Statistical analysis was performed with SPSS 13.0 (SPSS, Inc., Chicago, IL, USA). Comparisons between the groups were calculated with a *t*-test or one-way ANOVA for data with normal distribution and homoscedasticity, and the Wilcoxon’s rank-sum test was used for other data. Spearman’s correlation analysis was utilized to explore the correlation between *KDM6B* expression and *AR/CCND1* expression. The survival analysis was assessed by the log-rank test. *p* < 0.05 was considered statistically significant (two-sided).

## Results

### Expression profile and prognostic value of KDM6B in PCa

**Fig. 1:**
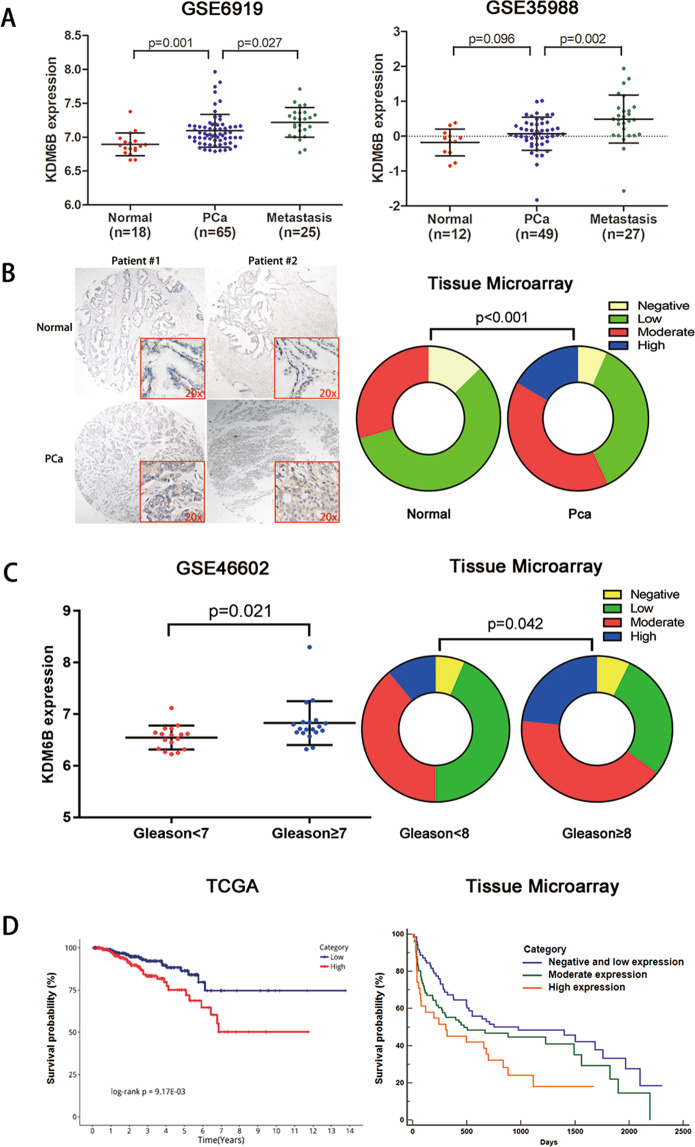
KDM6B identification and clinical relevance. **A** Public database indicates the differentiated expression of *KDM6B* mRNA among normal prostate tissue, prostate cancer tissue, and metastatic cancer tissue. **B** Tissue microarray data indicates elevated expression of KDM6B protein prostate cancer tissue. **C** The elevated KDM6B mRNA and protein expression levels in patients with a high Gleason Score are shown. **D** A Kaplan–Meier analysis of recurrence-free survival (mRNA, TCGA) and biochemical recurrence-free survival (protein, tissue microarray) in prostate cancer patients with differentiated KDM6B expression was performed (Note: Pca, prostate cancer; CRPC, castration-resistant prostate cancer. Gene expression data are presented as mean ± SD. Tissue microarray data are presented as percentage and detailed numbers of each degree are listed in Supplemental Tables.).

First, we utilized the public GEO databases and a tissue microarray to explore the mRNA and protein expression profiles of KDM6B in PCa (Fig. 1A). Representative specimens of the KDM6B immunohistochemistry results are presented in Supplemental Fig. 1A and the clinicopathological characteristics of patients enrolled in the tissue microarray are detailed in Supplemental Table 1. GEO:GSE6919 and GEO:GSE6752 datasets showed that *KDM6B* mRNA was expressed in all samples of normal prostate tissue, PCa tissue, and metastatic PCa tissue. Further, GEO:GSE35988 dataset indicated that the *KDM6B* mRNA expression rates in normal control, PCa, and metastatic PCa were 25%, 57%, and 70%, respectively. The tissue microarray data showed that KDM6B protein expression rates in normal control and PCa were 87% and 93%, respectively. At the mRNA level, GEO:GSE6919, GEO:GSE35988, and GEO:GSE6752 datasets indicated that *KDM6B* expression was upregulated in PCa compared to the expression in the normal control, and the expression was even higher in metastatic PCa (Fig. 1A and Supplemental Fig. 1B). The tissue microarray verified the elevated protein expression in PCa compared to the protein expression in the normal control (Fig. 1B and Supplemental Fig. 1B). In addition, GEO:GSE46602 dataset and the tissue microarray showed that KDM6B expression was higher in cases with a high Gleason score than the expression was in cases with a low Gleason score (Fig. 1C). The tissue microarray also found elevated KDM6B protein expression in patients with seminal vesicle invasion and nerve invasion (Supplemental Table 2). Furthermore, a survival analysis of the TCGA database showed that patients with high *KDM6B* mRNA expression experienced shorter recurrence-free survival (Fig. 1D). A univariate and multivariate survival analysis of the tissue microarray data verified the findings that patients with elevated KDM6B protein levels were associated with poor BCR-free survival (Fig. 1D and Supplemental Table 3). However, the KDM6B expression levels failed to predict the overall survival of PCa patients (Supplemental Fig. 1D).

### KDM6B plays an oncogenic role in PCa

**Fig. 2:**
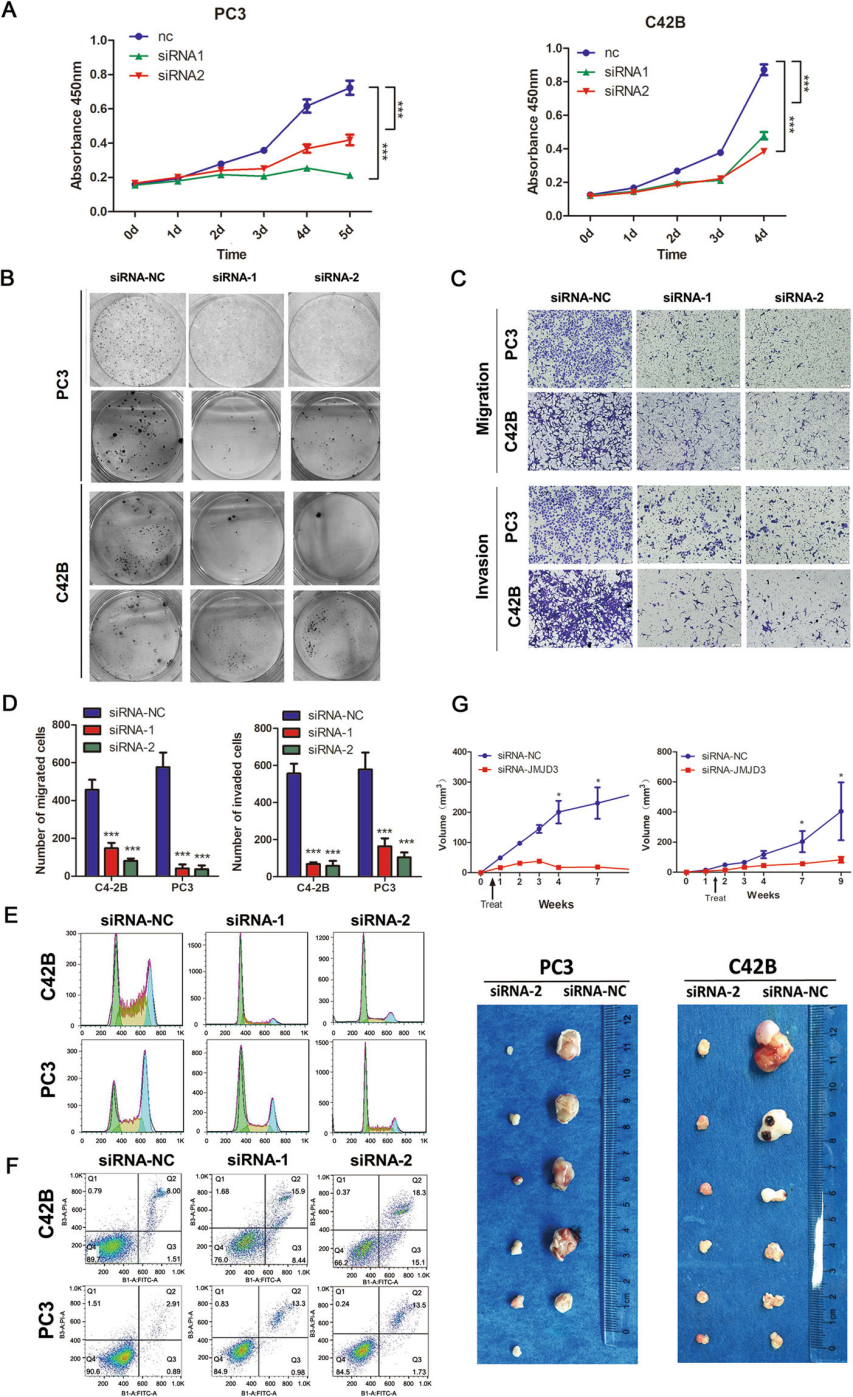
Cellular function and in vivo experiments. **A** The proliferation abilities of PC3 and C42B cells transfected with *KDM6B* siRNA are shown. Error bars means SD, *N* = 3 independent repeats. **B** The colony formation abilities of PC3 and C42B cells transfected with *KDM6B* siRNA are shown. **C**, **D** The knockdown of *KDM6B* by siRNA suppressed the migration and invasion abilities of PC3 and C42B cells. Error bars means SD, *n* = 3 independent repeats. **E** A cell cycle analysis of PC3 and C42B cells transfected with *KDM6B* siRNA was performed. **F** An apoptosis analysis of PC3 and C42B cells transfected with *KDM6B* siRNA was performed. **G** Nude mice were subcutaneously xenografted with PC3 and C42B cells, and were treated intratumorally with scrambled or *KDM6B* siRNA. The tumor growth curve and representative tumor images are shown (Note: AR, androgen receptor; TF, transcription factor; DHT, dihydrotestosterone; MDV3100, enzalutamide; DMSO, dimethylsulfoxide; WT, wild type; MUT, mutation.) Error bars mean SD, six mice in each group with one lost in PC3 cell line + negative control siRNA (*p < 0.05, ***p < 0.001).

To explore the potential roles of *KDM6B* in PCa, we constructed two siRNAs. The details of the siRNA sequence are presented in the Supplemental Materials and the knockdown (KD) efficiency was tested (Supplemental Fig. 2A, B). *KDM6B* KD significantly decreased the proliferation of PC3 and C42B cell lines according to a CCK-8 assay (Fig. 2A). In addition, *KDM6B* KD also inhibited the colony formation abilities of the PC3 and C42B cell lines (Fig. 2B). Furthermore, the migration and invasion abilities of the PC3 and C42B cell lines were reduced after *KDM6B* KD (Fig. 2C, D). Moreover, *KDM6B* KD significantly increased the sub-G0-G1 populations, decreased the sub-S-G2 populations, and induced more apoptosis in the PC3 and C42B cell lines (Fig. 2E, F).

To further test the effects of *KDM6B* KD on PCa growth in vivo, nude mice were subcutaneously implanted with PC3 and C42B cells, and the combination of in vivo-jetPEI delivery reagent and the *KDM6B* siRNA were applied to knock down *KDM6B* expression in xenograft (Supplemental Fig. 2C, D). No prior test and treatment were administrated to enrolled mice. One mouse in the PC3 + negative control group was dead due to unknown reason, whereas no significant adverse event was observed in other mice included in the analysis. The in vivo experiment showed *KDM6B* KD in the PC3 and C42B cell xenografts remarkably decreased the tumor growth velocity and volume (Fig. 2G).

### GSK-J4 inhibits the growth of PCa

**Fig. 3:**
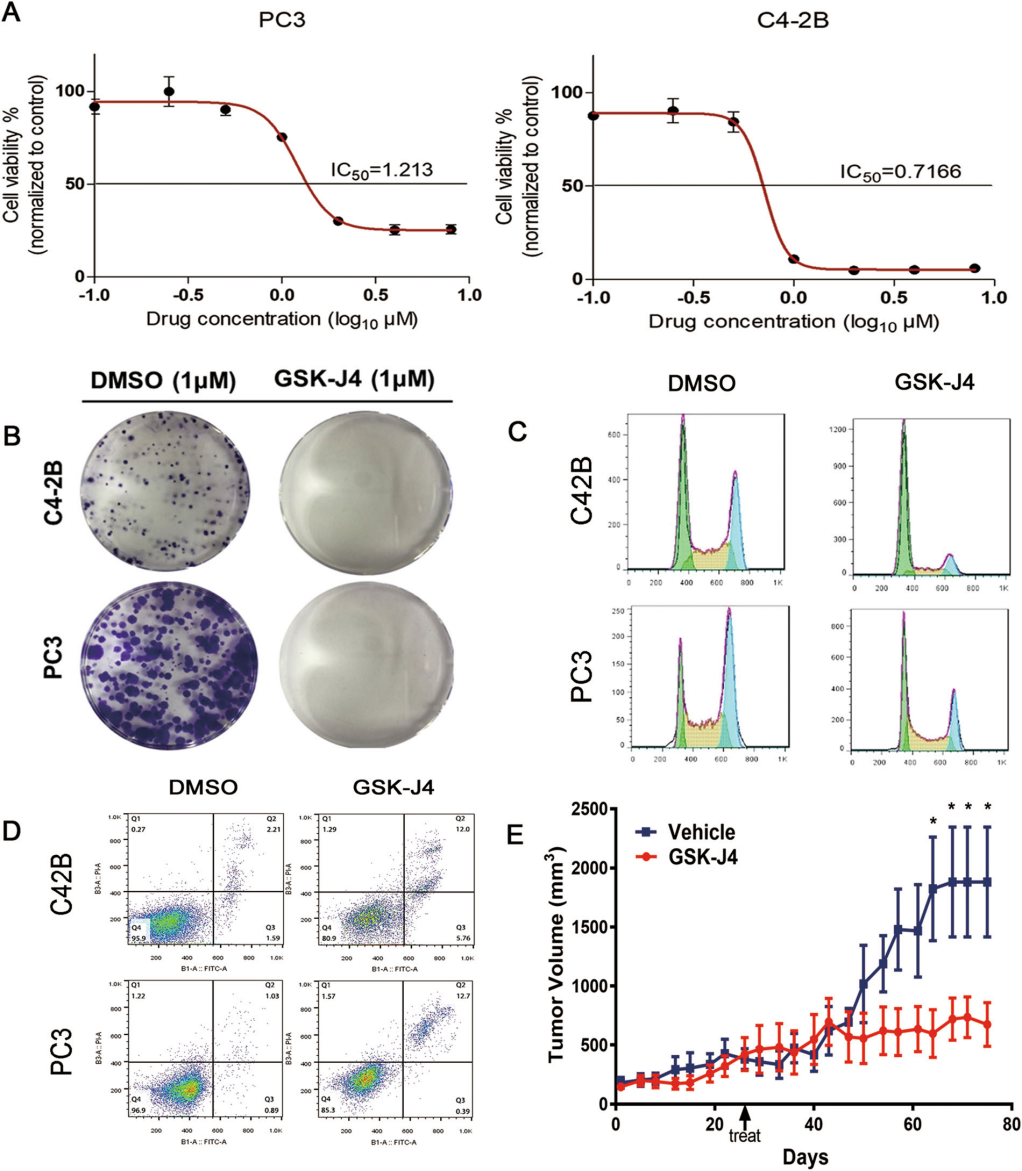
GSK-J4 inhibits cellular function and in vivo experiments. **A** The IC50 values of GSK-J4 for viability inhibition in PC3 and C42B cells are shown. Error bars means SD, *n* = 3 independent repeats. **B** The colony formation abilities of PC3 and C42B cells treated with GSK-J4 are shown. **C** A cell cycle analysis of PC3 and C42B cells treated with GSK-J4 was performed. **D** An apoptosis analysis of PC3 and C42B cells treated with GSK-J4 was performed. **E** The growth curve of subcutaneous xenograft of C42B cells treated with GSK-J4. Error bars mean SD, six mice in each group (*p < 0.05).

GSK-J4 administration in PCa cell lines revealed a dose-dependent inhibition of cellular viability and the growth rates were inhibited by 50% at concentrations of 1.213 μM and 0.7166 μM for the PC3 and C42B cell lines, respectively (Fig. 3A). In addition, GSK-J4 treatment inhibited the ability of the PC3 and C42B cell lines to form colonies (Fig. 3B). An analysis of the cell cycle distributions by flow cytometry revealed that there were increased levels of sub-G0-G1 populations and decreased levels of sub-S-G2 populations in PC3 and C42B cell lines that were treated with GSK-J4 (Fig. 3C). In addition, GSK-J4 also led to more apoptosis in the PC3 and C42B cell lines (Fig. 3D). Furthermore, the administration of GSK-J4 suppressed the tumor growth velocity and volume in a C42B mice tumor model (Fig. 3E).

### KDM6B is involved in the development of CRPC

**Fig. 4:**
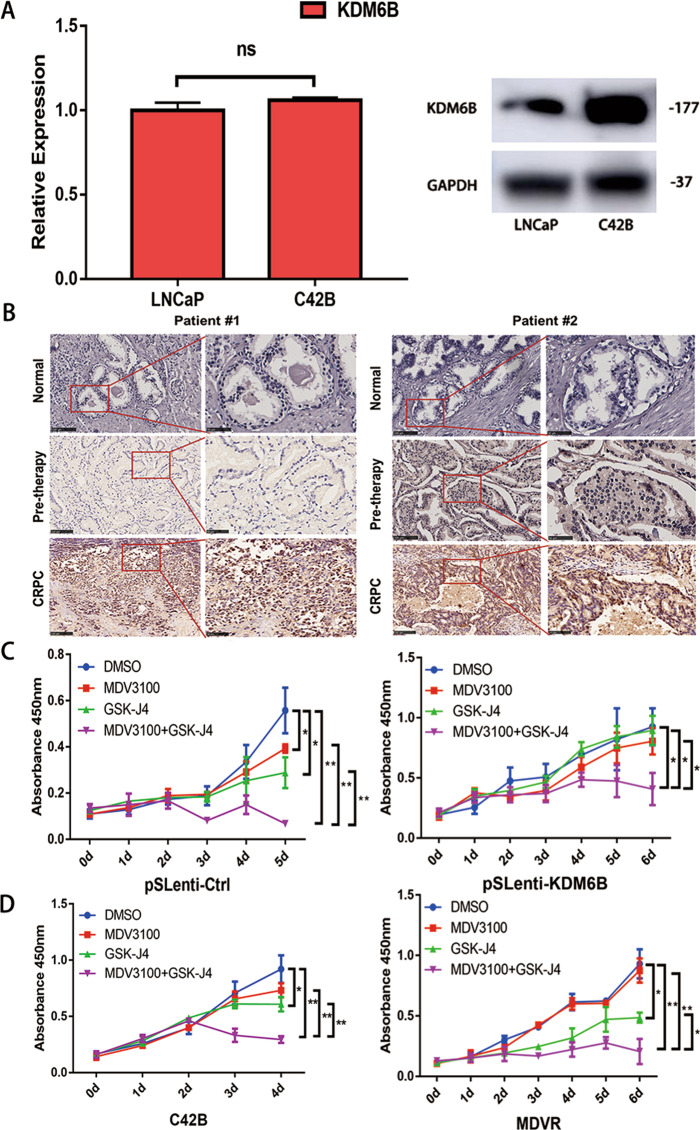
KDM6B is involved in the development of CRPC. **A** Comparison of KDM6B mRNA and protein expression between LNCaP and C42B cells. Error bars means SD, *n* = 3 independent repeats. **B** The immunochemistry results of KDM6B in normal prostate tissue, pretherapy prostate cancer tissue, and castration-resistant prostate cancer tissue are shown. **C** Single MDV3100 and GSK-J4 treatment failed to suppress the proliferation of pSLenti-*KDM6B*-transfected LNCaP cells, but the phenomenon could be terminated by the combination of MDV3100 plus GSK-J4. Error bars means SD, *n* = 3 independent repeats. **D** Combination of MDV3100 plus GSK-J4 was effective for CRPC C42B cells and MDV3100-resistant CRPC MDVR cells. Error bars mean SD, *n* = 3 independent repeats (ns: not significant, *p < 0.05, **p < 0.01; MDV3100, enzalutamide; DMSO, dimethylsulfoxide).

To explore the *KDM6B* expression profile in castration-resistant PCa (CRPC), we tested KDM6B mRNA and protein expression in hormone-sensitive LNCaP cells and castration-resistant C42B cells. Even though no significant difference of *KDM6B* mRNA expression was calculated between LNCaP and C42B cells, elevated KDM6B protein expression was observed in C42B cells (Fig. 4A). Then, we conducted immunohistochemistry on PCa specimens from two patients with consecutive normal, pretherapy, and CRPC specimens. Compared to those in the pretherapy PCa specimens, a sharp increase in the KDM6B protein expression levels were observed in the CRPC specimens (Fig. 4B). Next, pSLenti-Ctrl and pSLenti-*KDM6B* were transfected in LNCaP cells to construct negative control and *KDM6B*-overexpressed LNCaP cells (Supplemental Fig. 2E, F). We observed that combined MDV3100 (enzalutamide) and GSK-J4 treatment yielded higher inhibition efficiency than single MDV3100 and GSK-J4 treatment in pSLenti-Ctrl-transfected LNCaP cells (Fig. 4C). Intriguingly, single MDV3100 and GSK-J4 treatment failed to suppress the proliferation of LNCaP cells transfected with pSLenti-*KDM6B*, but the combination MDV3100 and GSK-J4 treatment could significantly inhibit its proliferation (Fig. 4C). In castration-resistant C42B cells and MDV3100-resistant CRPC MDVR cells, combination of MDV3100 and GSK-J4 treatment had higher proliferation inhibition efficiency than single MDV3100 or GSK-J4 treatment (Fig. 4D). These results showed that KDM6B is involved in the development of CRPC, and combination of MDV3100 plus GSK-J4 is effective for CRPC and MDV3100-resistant CRPC.

### ARs suppress KDM6B transcription

**Fig. 5:**
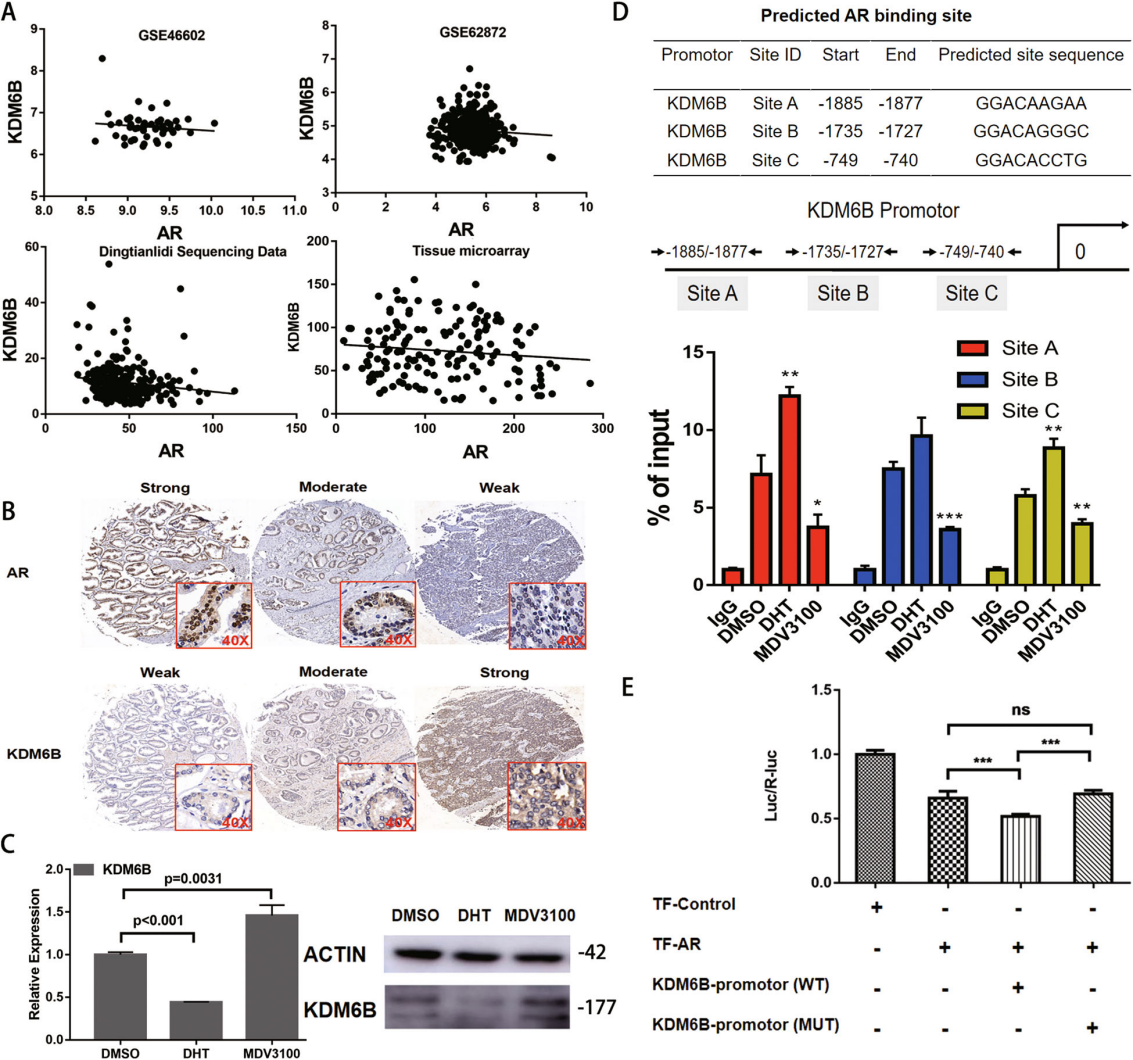
KDM6B expression is downregulated by ARs. **A** A correlation analysis between *KDM6B* and *ARs* using data from GEO:GSE46602 (*n* = 50, *p* = 0.83, *r* = -0.03), GEO:GSE62872 (*n* = 424, *p* = 0.18, *r* = −0.07), Dingtianlidi sequencing program (*n* = 271, *p* = 0.02, *r* = −0.18) and the tissue microarray (*n* = 156, *p* = 0.33, *r* = −0.08) was performed. **B** Representative images of the KDM6B and AR immunohistochemistry analysis indicate a negative correlation between KDM6B and ARs. **C** Comparisons of the KDM6B mRNA and protein expression levels among the DMSO, DHT, and MDV3100 treatments in the LNCaP cell line were performed by PCR and western blot analyses. Error bars means SD, *n* = 3 independent repeats. **D** Comparisons of the AR-binding levels to the *KDM6B* promoter among the DMSO, DHT, and MDV3100 treatments in the LNCaP cell line were performed using a ChIP-PCR analysis. Error bars means SD, *n* = 3 independent repeats. **E** Dual-luciferase reporter assay was used to verify that AR directly regulated *KDM6B* transcription by 3′-UTR. Error bars means SD, *n* = 3 independent repeats (Note: AR, androgen receptor; DHT, dihydrotestosterone; MDV3100, enzalutamide; DMSO, dimethylsulfoxide) (ns: not significant, *p < 0.05, **p < 0.01, ***p < 0.001).

When analyzing the KDM6B expression data in the tissue microarray, we observed a phenomenon in which patients with high serum PSA (≥20 ng/ml) had lower KDM6B protein expression levels than those in patients with low serum PSA (<20 ng/ml) (Supplemental Fig. 3); this inspired us to determine whether there is an association between *ARs* and *KDM6B*. Although the GEO:GSE46602 dataset, the GEO:GSE62872 dataset, our Dingtianlidi sequencing data, and the tissue microarray data indicated a negative correlation between *ARs* and *KDM6B*, statistical significance was only calculated for the Dingtianlidi sequencing dataset (Fig. 5A, B). Then, we applied DHT (dihydrotestosterone, AR activator) and MDV3100 (enzalumide, AR inhibitor) to treat the AR-postitive LNCaP cell line. DHT decreased the mRNA and protein expression levels of KDM6B, whereas MDV3100 elevated the mRNA and protein expression levels of KDM6B (Fig. 5C). To provide mechanistic support for the potential AR transcriptional regulation of *KDM6B*, we performed quantitative ChIP assays to determine whether ARs can directly bind to the *KDM6B* promoter. Multiple putative AR-binding sites were predicted within 2000 bp upstream of the *KDM6B* transcription start site and three binding sites were confirmed. Compared to that in the control DMSO group, DHT increased the level of ARs binding to the *KDM6B* promoter, which might then lead to the suppression of *KDM6B* expression. In contrast, MDV3100 decreased these binding abilities, which might then lead to an increase in *KDM6B* expression (Fig. 5D). For further confirmation, we constructed luciferase reporters containing the WT *KDM6B* promoter sequence (WT) and MUT in the AR-binding sites at the 3′-untranslated region of Rluc. We found that AR reduced the luciferase activities of the WT reporter vector but not mutant reporter vector (Fig. 5E). These data demonstrated that AR could decrease *KDM6B* transcription by directly binding its promotor.

### KDM6B prompts CCND1 expression via demethylating H3K27me3

**Fig. 6:**
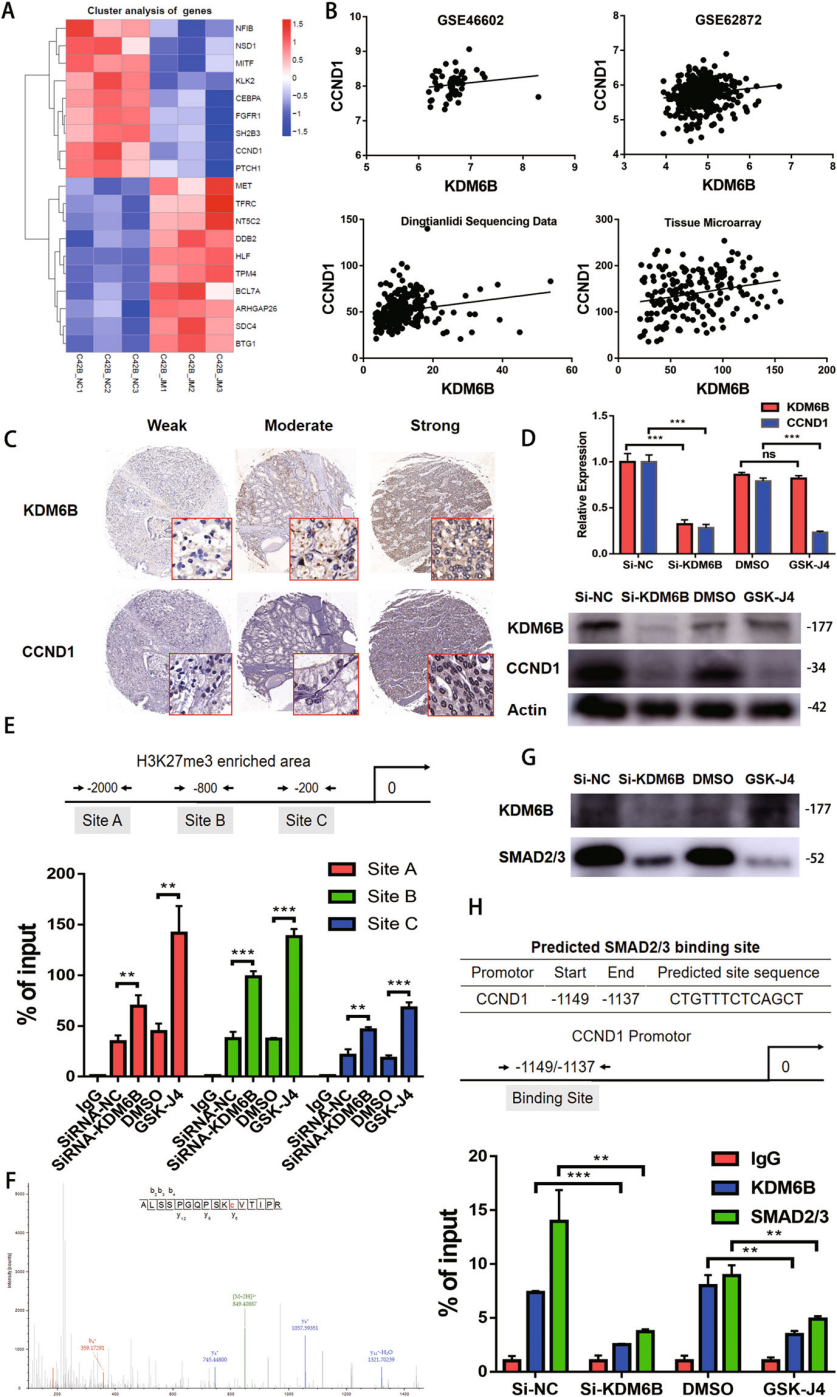
KDM6B demethylates H3K27me3 on the promoter of CCND1 and cooperates with smad2/3 to prompt the expression of CCND1. **A** A supervised hierarchical clustering of the genes differentially expressed between the C42B wild-type samples and the *KDM6B* depletion C42B samples is shown. **B** A correlation analysis between *KDM6B* and *CCND1* using data from GEO:GSE46602 (*n* = 50, *p* = 0.05, *r* = 0.28), GEO:GSE62872 (*n* = 424, *p* = 0.001, *r* = 0.15), Dingtianlidi sequencing program (*n* = 271, *p* < 0.001, *r* = 0.34) and the tissue microarray (*n* = 176, *p* = 0.002, *r* = 0.23) was performed. **C** Representative images of the KDM6B and CCND1 immunohistochemistry results indicate a positive correlation between KDM6B and CCND1. **D** Comparisons of the CCND1 mRNA and protein expression levels among the siRNA-NC, siRNA-KDM6B, DMSO, and GSK-J4 treatments in the PC3 cell line were performed by PCR and western blot analyses. Error bars mean SD, *n* = 3 independent repeats. **E** A ChIP-PCR analysis demonstrates that the depletion and inhibition of KDM6B increases H3K27me3 on the *CCND1* promoter in the PC3 cell line. Error bars mean SD, *n* = 3 independent repeats. **F** Immunoprecipitation-based mass spectrometry revealed the interaction between KDM6B and Smad2/3. **G** An immunoprecipitation analysis indicates that treatments with siRNA-*KDM6B* and GSK-J4 reduce the amount of interaction between KDM6B and Smad2/3. **H** Comparisons of the KDM6B and Smad2/3-binding levels to the *CCND1* promoter among the siRNA-NC, siRNA-*KDM6B*, DMSO, and GSK-J4 treatments in the PC3 cell line were performed using a ChIP-PCR analysis. Error bars mean SD, *n* = 3 independent repeats (Note: DMSO, dimethylsulfoxide, ns: not significant, **p < 0.01, ***p < 0.001).

To explore the downstream molecular mechanism of KDM6B in PCa, RNA sequencing was conducted to identify differentiated gene expression between the C42B WT cells and the C42B *KDM6B* KD cells. A series of genes were downregulated or upregulated in the C42B *KDM6B* KD cells (Fig. 6A) and *CCND1* was chosen as the target of future research. All of the GEO:GSE46602 dataset, GEO:GSE62872 dataset, Dingtianlidi sequencing data (http://bigd.big.ac.cn/gsa-human/; accession: PRJCA001124), and the tissue microarray data indicated that there was a positive correlation trend between KDM6B and CCND1 expression, and the statistical significance was not calculated for the GEO:GSE46602 dataset (*p* = 0.05); this may be due to the limited sample size (Fig. 6B, C). Quantitative PCR and western blot analyses indicated significantly reduced KDM6B and CCND1 mRNA and protein expression levels in PC3 *KDM6B* KD cells (Fig. 6D). The administration of GSK-J4 also decreased the CCND1 mRNA and protein expression levels in the PC3 cell line (Fig. 6D). Then, a quantitative ChIP experiment was conducted to test whether the *CCND1* expression level was correlated with H3K27me3 modifications at the promoter in PC3 cells. After the siRNA-mediated KD and the GSK-J4-mediated inhibition of KDM6B, the abundance of H3K27me3 at the *KDM6B* promoter was significantly increased (Fig. 6E) and this led to the suppression of *CCND1* expression. To search for the transcription factor that cooperates with KDM6B, an immunoprecipitation assay targeting KDM6B and a subsequent mass spectrometry analysis were conducted. The mass spectrum identified that smad2/3 binds to KDM6B (Fig. 6F and Supplemental Fig. 4). After the silencing and inhibition of KDM6B in PC3 cells, the level of the binding between smad2/3 and KDM6B decreased (Fig. 6G), which indicated that the binding of smad2/3 to KDM6B was correlated with KDM6B expression and activity. Next, the quantitative ChIP assay showed that the siRNA-mediated KD and the GSK-J4-mediated inhibition of KDM6B significantly decreased the binding of KDM6B and smad2/3 to the promotor of CCND1 (Fig. 6H). In summary, KDM6B and smad2/3 cooperatively regulate *CCND1* transcription. KDM6B demethylates H3K27me3 to facilitate *CCND1* transcription, and smad2/3 works as the transcriptional factor during the process.

## Discussion

In this study, we demonstrate five major findings. First, the KDM6B mRNA and protein levels were found to be upregulated in PCa, especially in metastatic PCa and in CRPC. In addition, the overexpression of KDM6B is correlated with the early recurrence of PCa. Second, KDM6B could promote the proliferation, migration, and invasion of PCa cells in vitro and could prompt the growth of PCa in vivo. Third, GSK-J4 could decrease the viability of PCa cells, inhibit PCa cell proliferation, block the cell cycle and prompt apoptosis in vitro, and inhibit the growth of PCa in vivo. Fourth, *KDM6B* expression is negatively regulated by ARs. Fifth, KDM6B demethylates H3K27 at the *CCND1* promoter region and cooperates with smad2/3 to prompt the expression of *CCND1*.

A previous study searched the Oncomine database and one set of data showed that the *KDM6B* mRNA expression rates in benign prostate hyperplasia, PCa, and metastatic PCa were 4.8%, 16%, and 55%, respectively^25^. The average *KDM6B* expression is limited in benign prostate hyperplasia, upregulated in PCa, and even higher in metastatic PCa^25^. At the protein level, their western blot analysis indicated that two benign prostate hyperplasia cases had no KDM6B expression, but KDM6B was detected in three out of four PCa tissue samples^25^. Of note, the case without KDM6B expression had a Gleason score of 3 + 3, whereas the positive cases had Gleason scores of at least 4 + 4^25^. In contrast to the above report, we found that KDM6B was commonly expressed in benign prostate tissue and in PCa at the mRNA and protein levels. And a trend of gradually increasing expression from benign prostate tissue to PCa to metastatic PCa was observed in this study. In addition, KDM6B expression in the low PSA group (<20) is higher than that in the high PSA group (≥20), which causes us to wonder whether there is a negative correlation between *ARs* and *KDM6B*. Furthermore, a univariate and multivariate survival analysis showed that overexpressed KDM6B was correlated with the early recurrence of PCa, but no correlation was found between KDM6B expression and overall survival. KDM6B has the potential to independently predict the early recurrence of PCa.

Previous studies have reported the oncogenic role of KDM6B in various malignant tumors^14,15,18,29,30^. On the other hand, it was reported that KDM6B-induced INK4a/P16 expression benefited the survival of cervical carcinoma^19^. Another study showed that a KDM6B insufficiency could promote the progression of pancreatic carcinoma by decreasing C/EBPα expression^20,21^. Moreover, a KDM6B deficiency with a subsequent H3K27me3 enrichment promoted the dedifferentiation of hypoxic^V600E^ BRAF melanoma cells and then led to BRAF inhibitor resistance^31^. These observations implicate the role of KDM6B in the progression of malignant tumors depending on tumor cell types. In this study, we found that the siRNA-mediated depletion of *KDM6B* inhibited the proliferation, migration, and invasion abilities of the C42B and PC3 cell lines. After the depletion of *KDM6B*, a very large proportion of the C42B and PC3 cells was blocked at the G1 phase and more apoptosis was observed. In addition, the KD of *KDM6B* inhibited the growth of PCa in vivo. These evidences indicated that *KDM6B* prompted the development and progression of PCa.

At present, GSK-J4 was reported to be the most useful tool for the inhibition of KDM6B demethylase activity^32^. Applying GSK-J4 to inhibit KDM6B could result in the growth inhibition of different cancer cell lines, including T-ALL and glioma^18,29^. In PCa, GSK-J4 could inhibit the proliferation of the C42B and PC3 cell lines, and the inhibition was demonstrated to be dose-dependent with 50% growth inhibition occurring at concentrations of 1.213 µM and 0.7166 µM for the PC3 and C42B cell lines, respectively. An analysis of the cell cycle distribution revealed that the G1 phase was the most increased cell cycle stage. In addition, GSK-J4 could also lead to greater levels of apoptosis in the C42B and PC3 cell lines, and could strongly inhibit PCa growth in vivo. Previous researches and our study, which illuminate the antitumoral effect of GSK-J4 by inhibiting KDM6B activity, indicate that a KDM6B-based target therapy is emerging as a promising cancer therapy for various tumors, including PCa.

Next, we focused on whether KDM6B promotes the development of CRPC. Compared to hormone-sensitive LNCaP cells, elevated KDM6B protein expression was found in castration-resistant C42B cells. In addition, we observed gradual increase of KDM6B expression from benign prostate tissue to PCa to CRPC. Of note, the KDM6B expression rise from PCa to CRPC was much greater than that from benign prostate tissue to PCa, which indicates that KDM6B might play a vital role in the development of CRPC. Further, MDV3100 administration failed to suppress the proliferation of LNCaP cells with KDM6B overexpression, whereas the combination of MDV3100 and GSK-J4 potently inhibited the growth of these cells. The above evidence indicates that KDM6B might involve in promoting PCa development after AR pathway was inhibited and the interaction between KDM6B and AR pathway need to be revealed. Compared to single MDV3100 or GAK-J4 treatment, the proliferation of castration-resistant C42B cells and MDV3100-resistant MDVR cell was significantly inhibited. It is reasonably believed that MDV3100 and GSK-J4 play a synergistic role in treating CRPC. Co-target AR and KDM6B could be a promising treatment for CRPC and MDV3100-resistant CRPC.

KDM6B is an inducible histone demethylase and its expression was shown to be mediated by NF-κB and signal transducer and activator of transcription 3 in previous reports^33,34^. As mentioned above, we found a negative association between KDM6B and PSA. Then, we speculated whether KDM6B expression could be induced or repressed by ARs. Therefore, we searched the GEO database and two sets of data (GSE46602 and GSE62872), and our Dingtianlidi sequencing data showed a negative correlation between *KDM6B* and *ARs* at the mRNA level. Although no statistically significant correlation between KDM6B protein expression and AR protein expression was calculated from the tissue microarray, a negative trend was observed. In reality, the H-score, applied to assess the immunohistochemistry results of the study, cannot provide enough precision to assess the protein expression levels. This may be the reason why no statistical significance was calculated when we observed a negative correlation between KDM6B and ARs at the protein level. To verify this finding, DHT and MDV3100 were applied to treat LNCaP cells. DHT decreased the expression of KDM6B mRNA and protein, whereas KDM6B mRNA and protein expression was increased by MDV3100 treatment. Further, a ChIP analysis revealed that DHT treatment increased the direct binding of ARs to the *KDM6B* promoter, and that MDV3100 reduced the direct binding of ARs to the *KDM6B* promoter. Dual-luciferase reporter assay also verified that ARs could decrease *KDM6B* transcription by directly binding its promotor. These evidence indicates that ARs play an inhibitory role in the transcription of *KDM6B*^35,36^. As AR activity loss is a common phenomenon in CRPC^36^, this relieves the repression of ARs on *KDM6B* transcription. Then, the accumulation of KDM6B facilitates the transcription of downstream oncogenic molecules in PCa; in turn, this prompts the conversion of PCa to CRPC.

The study revealed that *KDM6B* has great effects on the biological function of PCa, but the downstream mechanism is still unknown. To solve this issue, RNA sequencing was applied to compare the differences in gene expression between the C42B WT sample and the C42B *KDM6B* depletion sample. Compared to the C42B WT sample, a series of genes was upregulated or downregulated in the C42B *KDM6B* depletion sample, such as the upregulation of *CCND1*, *PTCH1*, *SH2B3*, and *FGFR1*, and the downregulation of *MET*, *TFRC*, and *NT5C2*. As *CCND1* was reported to have the functions of cell growth regulation, cell migration control, DNA repair, and mitochondrial activity modulation^37,38^, we chose *CCND1* as the focus of our downstream research. Two sets of GEO data, our Dingtianlidi sequencing data, and the tissue microarray data verified the positive correlation between *KDM6B* and *CCND1*. Furthermore, the CCND1 mRNA and protein expression level reduction was observed after the depletion and inhibition of KDM6B. ChIP-PCR demonstrated that the depletion and inhibition of KDM6B could increase histone H3K27me3 on the *CCND1* promoter. Additionally, an immunoprecipitation-based mass spectrometry analysis revealed an interaction between KDM6B and smad2/3. We also observed that the amount of the interaction between smad2/3 and KDM6B was reduced after the depletion and inhibition of KDM6B. Further, ChIP-PCR analysis showed that the depletion and inhibition of KDM6B reduced the binding of KDM6B and smad2/3 to the *CCND1* promoter. Together, these data reveal that KDM6B binds to the promoter regions of the *CCND1* gene, lessens H3K27me3 occupancy, and increases *CCND1* expression; in turn, this causes the progression of PCa.

In summary, we systemically used clinical data, in vitro cellular biological studies, and in vivo mouse models to reveal the oncogenic role of *KDM6B* in PCa. KDM6B could serve as a predictor for the early recurrence of PCa. In addition, GSK-J4, an inhibitor of KDM6B, could suppress the vitality and progression of PCa and cooperate with MDV3100 to suppress CRPC and MDV3100-resistant CRPC, it can serve as a promising therapeutic for the treatment of PCa. Furthermore, our study reveals the upstream and downstream mechanisms of KDM6B in PCa. We first report that ARs decrease the transcription of *KDM6B* and we report that KDM6B demethylates H3K27me3 on the promoter of *CCND1* and cooperates with smad2/3 to prompt the expression of *CCND1*.

## Supplementary information

Supplemental Figure Legends

Supplemental Fig.1

Supplemental Fig.2.tif

Supplemental Fig.3

Supplemental Fig.4

Supplemental Tables

siRNA and Primers used in the study

mass spectrometric analysis

The details of RNA sequencing and data processing

Dual-luciferase reporter assay report

C4-2B authentication

Lncap authentication

PC-3 authentication
