# Supplementary material for: KDM6B is an androgen regulated gene and plays oncogenic roles by demethylating H3K27me3 at cyclin D1 promoter in prostate cancer
Source: Cell Death Dis. 2021 Jan 6;12(1):2. doi: 10.1038/s41419-020-03354-4 (PMC7791132; doi:10.1038/s41419-020-03354-4)
Supplement: Supplementary file 1 — Supplemental Figure Legends [file 41419_2020_3354_MOESM1_ESM.docx]

**Supplemental Figure Legends**

**Fig. 1** KDM6B identification and clinical relevance

A: Representative specimens demonstrate the negative, low, moderate and high expression levels of the KDM6B protein.

B: Tissue microarray and public database data indicate the increased expression of the KDM6B protein (tissue microarray) in prostate cancer and the increased expression of the mRNA (GSE6752) in metastatic prostate cancer.

C: The Kaplan-Meier analysis results of the overall survival of prostate cancer patients were categorized by the low and high KDM6B expression levels from the TCGA database.

**Fig. 2** The designed siRNA efficiently knocked down KDM6B mRNA (A and C) and protein (B and D) expression in cells and xenograft, and transfection of pSLenti-KDM6B in LNCaP cells increased KDM6B mRNA (E) and protein (F) expression.

**Fig. 3** Comparison of KDM6B protein expression between patients with low (<20 ng/ml) and high (≥20 ng/ml) PSA expression using H-scores.

(Note: PSA, prostate specific antigen.)

**Fig. 4** Representative peptide fragments of Smad 2/3 revealed by immunoprecipitation-based mass spectrometry.
