# Supplementary figures and images for: KDM6B is an androgen regulated gene and plays oncogenic roles by demethylating H3K27me3 at cyclin D1 promoter in prostate cancer
Source: Cell Death Dis. 2021 Jan 6;12(1):2. doi: 10.1038/s41419-020-03354-4 (PMC7791132; doi:10.1038/s41419-020-03354-4)

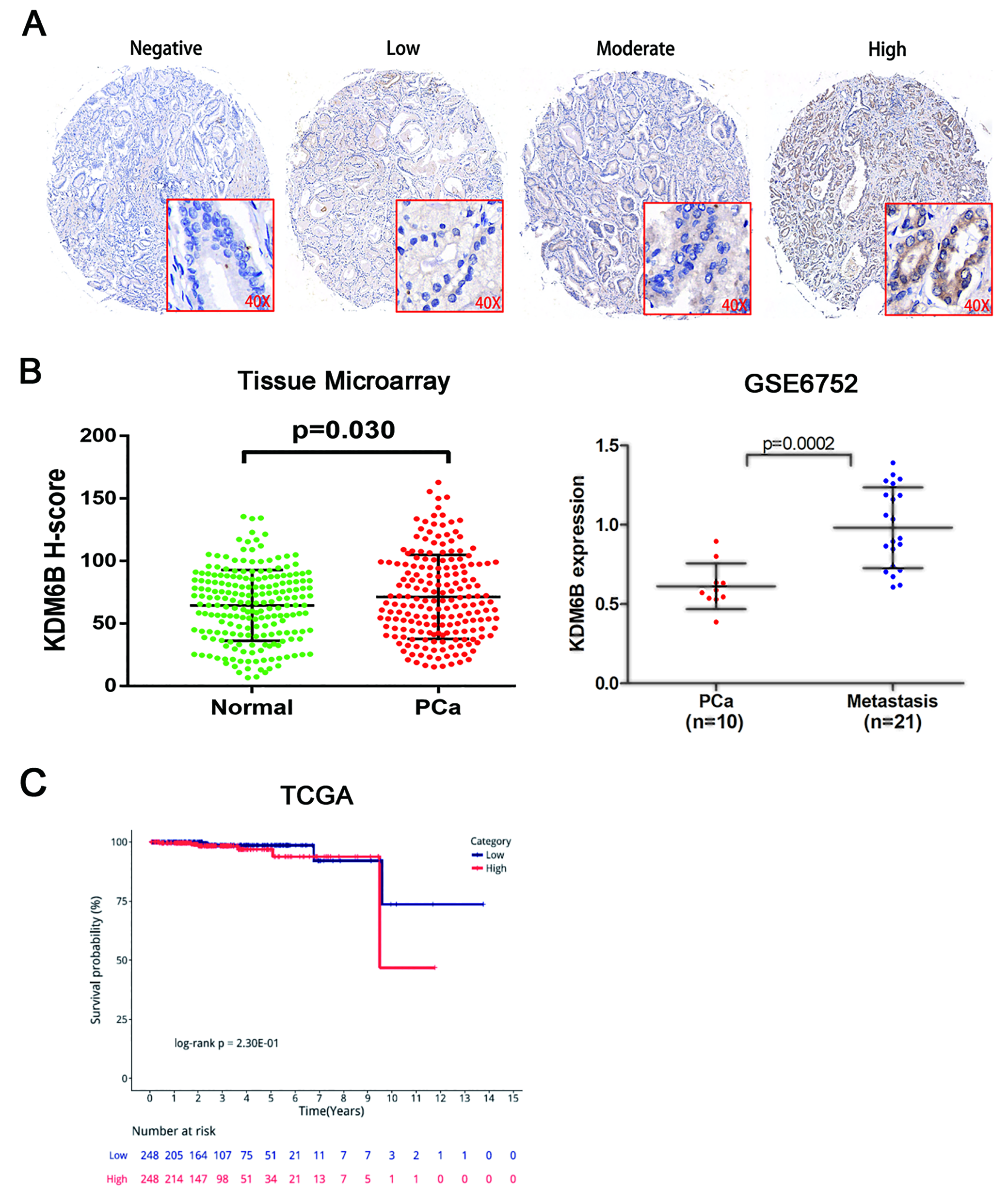

Supplement: Supplementary file 2 — Supplemental Fig.1 [file 41419_2020_3354_MOESM2_ESM.tif]

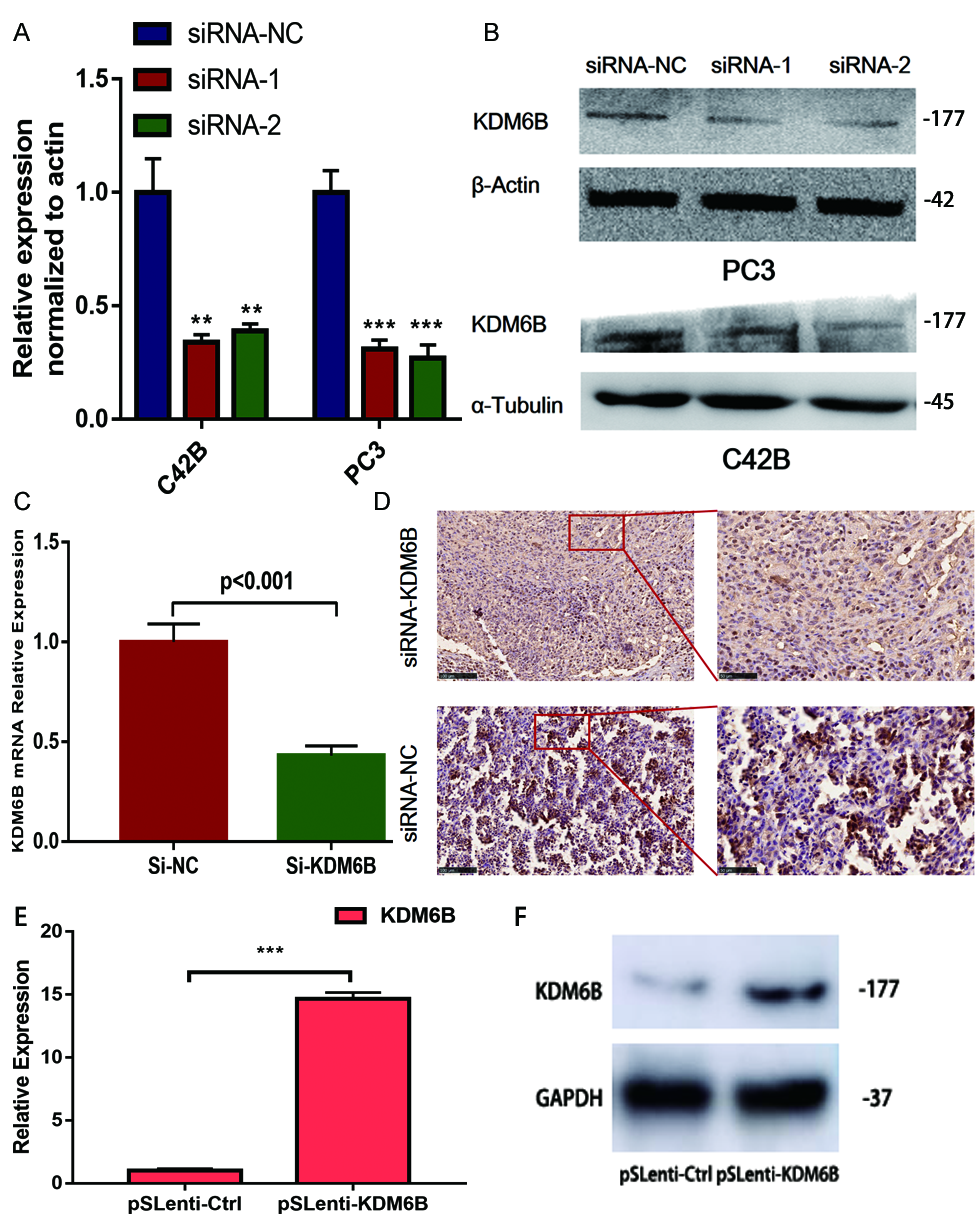

Supplement: Supplementary file 3 — Supplemental Fig.2.tif [file 41419_2020_3354_MOESM3_ESM.tif]

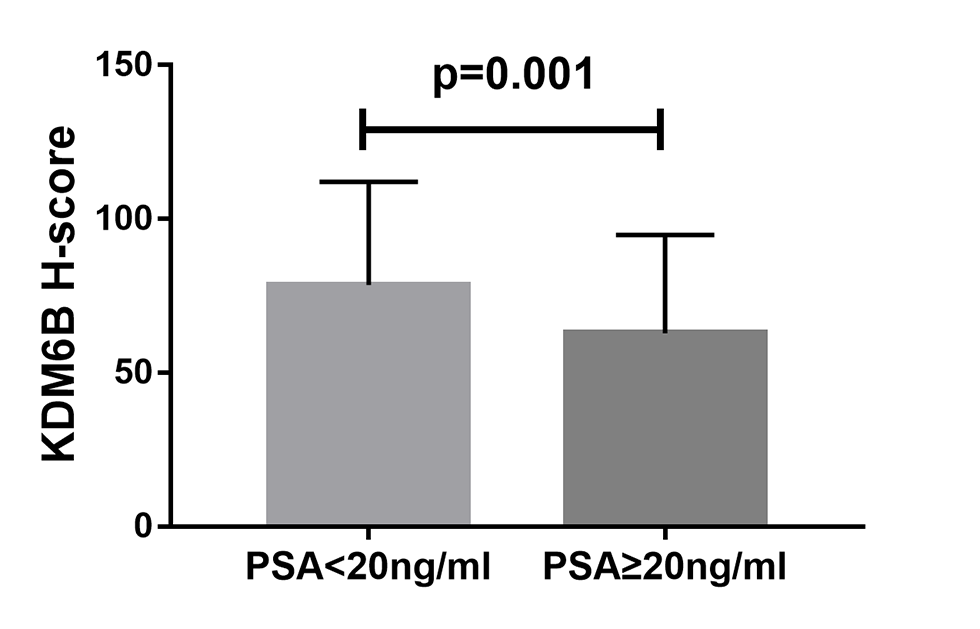

Supplement: Supplementary file 4 — Supplemental Fig.3 [file 41419_2020_3354_MOESM4_ESM.tif]

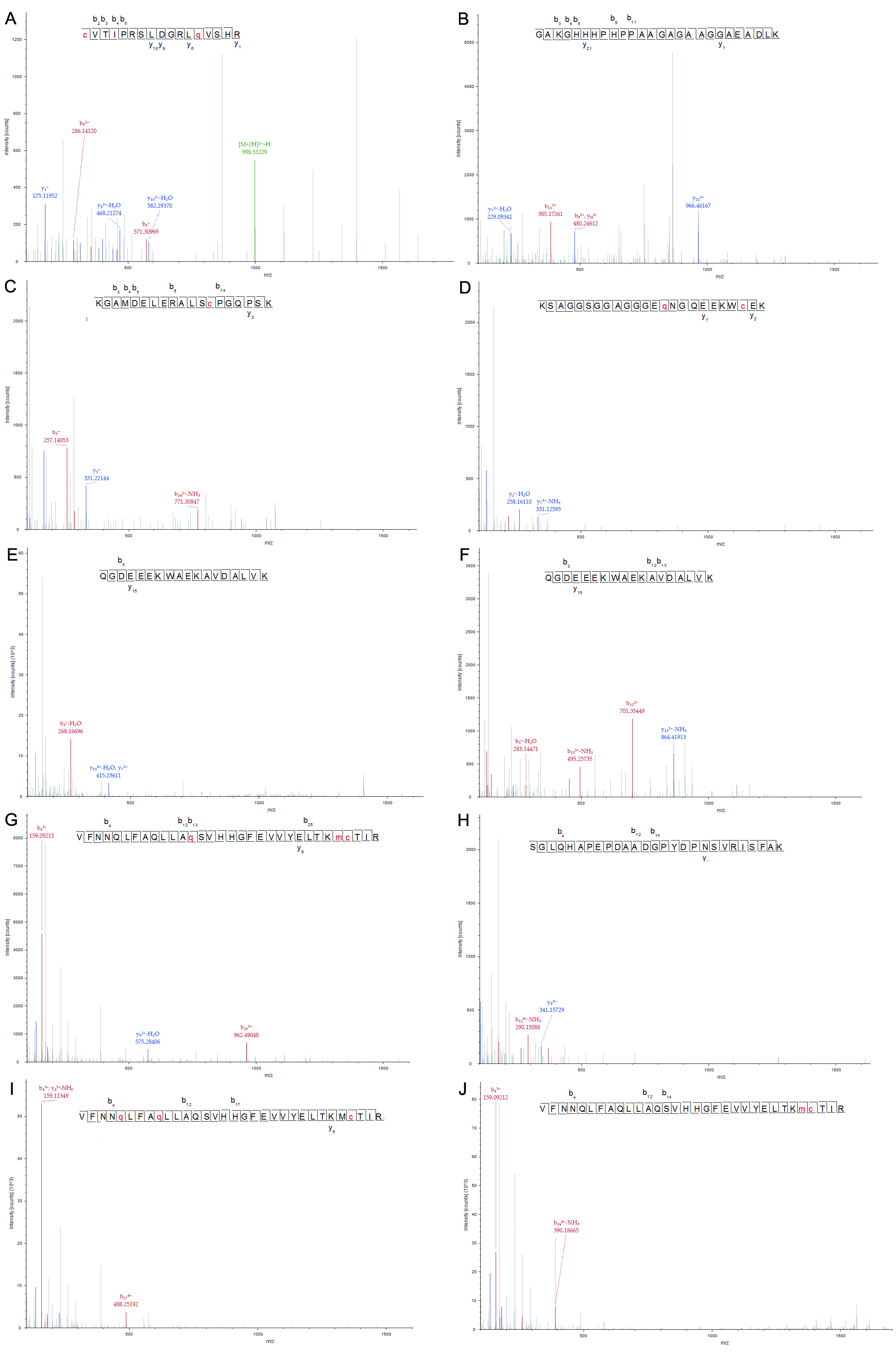

Supplement: Supplementary file 5 — Supplemental Fig.4 [file 41419_2020_3354_MOESM5_ESM.tif]
