# Supplementary material for: KDM6B is an androgen regulated gene and plays oncogenic roles by demethylating H3K27me3 at cyclin D1 promoter in prostate cancer
Source: Cell Death Dis. 2021 Jan 6;12(1):2. doi: 10.1038/s41419-020-03354-4 (PMC7791132; doi:10.1038/s41419-020-03354-4)
Supplement: Supplementary file 6 — Supplemental Tables [file 41419_2020_3354_MOESM6_ESM.docx]

Table 1 Pathological and clinical characteristics of the enrolled prostate cancers

| Variable | Prostate cancer  (n=202) | BPH  (n=29) | p |
| --- | --- | --- | --- |
| Age(years) | 65.9±11.0 | 68.8±7.3 | 0.166 |
| BMI | 24.32±2.68 | 24.62±3.24 | 0.579 |
| Pre-operation PSA(ng/ml) | 42.66±105.3  (n=200) | 7.16±4.50  (n=28) | 0.076 |
| Pre-operation f/tPSA(ng/ml) | 0.11±0.09  (n=134) | 0.24±0.10  (n=19) | <0.001 |
| pT category (AJCC 2002) |  |  |  |
| pT2 | 112(55.45%) |  |  |
| pT3 | 77(38.12%) |  |  |
| PT4 | 7(3.47%) |  |  |
| NA | 6(2.97%) |  |  |
| Gleason grade |  |  |  |
| ≤3+3 | 10(4.95%) |  |  |
| 3+4 | 64(31.68%) |  |  |
| 4+3 | 33(16.34%) |  |  |
| ≥4+4 | 95(47.03%) |  |  |
| pN category |  |  |  |
| pN0 | 121(59.90%) |  |  |
| pN+ | 42(20.79%) |  |  |
| NA | 39(19.31%) |  |  |
| Prostate capsule invasion |  |  |  |
| Negative | 119(58.91%) |  |  |
| Positive | 77(38.12%) |  |  |
| NA | 6(2.97%) |  |  |
| Seminal vesicle invasion |  |  |  |
| Negative | 147(72.77%) |  |  |
| Positive | 49(24.26%) |  |  |
| NA | 6(2.97%) |  |  |
| Surgical margin |  |  |  |
| Negative | 106(52.48%) |  |  |
| Positive | 90(44.55%) |  |  |
| NA | 6(2.97%) |  |  |
| Nerve invasion |  |  |  |
| Negative | 104(51.49%) |  |  |
| Positive | 92(45.54%) |  |  |
| NA | 6(2.97%) |  |  |

Table 2 KDM6B expression among variables.

| Variable | NO. of Cases | KDM6B IHC Result | | | | P-value |
| --- | --- | --- | --- | --- | --- | --- |
|  |  | Negative | Low | Moderate | High |  |
| Tissue type |  |  |  |  |  | <0.001 |
| Prostate cancer | 209 | 14 | 76 | 84 | 35 |  |
| Benign prostate tissue | 227 | 29 | 131 | 67 | 0 |  |
| pT stage |  |  |  |  |  | 0.061 |
| pT2 | 116 | 8 | 50 | 46 | 12 |  |
| pT3/4 | 88 | 6 | 26 | 36 | 20 |  |
| Gleason grade |  |  |  |  |  | 0.042 |
| <4+4 | 110 | 7 | 48 | 43 | 12 |  |
| ≥4+4 | 99 | 7 | 28 | 41 | 23 |  |
| Pre-operation PSA level (ng/ml) |  |  |  |  |  | 0.568 |
| <20 | 110 | 7 | 42 | 46 | 15 |  |
| ≥20 | 97 | 7 | 32 | 38 | 20 |  |
| pN category |  |  |  |  |  | 0.091 |
| pN0 | 124 | 10 | 55 | 44 | 15 |  |
| pN+ | 43 | 1 | 13 | 19 | 10 |  |
| Prostate capsule invasion |  |  |  |  |  | 0.057 |
| Negative | 120 | 9 | 51 | 48 | 12 |  |
| Positive | 83 | 5 | 25 | 34 | 19 |  |
| Seminal vesicle invasion |  |  |  |  |  | 0.011 |
| Negative | 150 | 10 | 63 | 61 | 16 |  |
| positive | 53 | 4 | 13 | 21 | 15 |  |
| Surgical margin |  |  |  |  |  | 0.099 |
| Negative | 108 | 7 | 47 | 43 | 11 |  |
| Positive | 95 | 7 | 29 | 39 | 20 |  |
| Nerve invasion |  |  |  |  |  | 0.026 |
| Negative | 108 | 5 | 45 | 48 | 10 |  |
| Positive | 95 | 9 | 31 | 34 | 21 |  |

Table 3 Cox’s univariate and multivariate analysis of biochemical recurrence-free time according to KDM6B expression.

|  | BCR-free survival  (Protein, Tissue Microarray) | |
| --- | --- | --- |
|  | RR(95%CI) | P |
| Univariate analysis |  |  |
| KDM6B |  |  |
| Negative and Low | 1 |  |
| Moderate and High | 1.583 (1.071-2.341) | 0.021 |
| Multivariate analysis |  |  |
| KDM6B |  |  |
| Negative and Low | 1.000 |  |
| Moderate and High | 1.639(1.051-2.558) | 0.029 |
| pT stage |  |  |
| Low(pT2) | 1 |  |
| High(pT3-4) | 1.672(0.980-2.851) | 0.059 |
| pN stage |  |  |
| Negative | 1 |  |
| Positive | 2.033(1.243-3.325) | 0.005 |
| Gleason score |  |  |
| Low(<4+4) | 1 |  |
| High(≥4+4) | 1.525(0.925-2.514) | 0.098 |
| PSA level |  |  |
| Low(<20ng/ml) | 1.000 |  |
| High(≥20ng/ml) | 1.403(0.881-2.233) | 0.153 |
