# Supplementary material for: KDM6B is an androgen regulated gene and plays oncogenic roles by demethylating H3K27me3 at cyclin D1 promoter in prostate cancer
Source: Cell Death Dis. 2021 Jan 6;12(1):2. doi: 10.1038/s41419-020-03354-4 (PMC7791132; doi:10.1038/s41419-020-03354-4)
Supplement: Supplementary file 7 — siRNA and Primers used in the study [file 41419_2020_3354_MOESM7_ESM.docx]

# Supplemental information

siRNA sequence of KDM6B

Primers for proteins tested in the study

Primers of AR on KDM6B promotor in ChIP-PCR

Primers of Smad2/3 on CCND1 promotor in ChIP-PCR

Primers of H3K27mes on CCND1 promotor in ChIP-PCR

siRNA sequence of KDM6B

| siRNA 1 | Sense (5‘-3‘) | CCUGCACUUUCUCGGACCA(dTdT) |
| --- | --- | --- |
|  | Antisense(5‘-3‘) | UGGUCCGAGAAAGUGCAGG(dTdT) |
| siRNA 2 | Sense (5‘-3‘) | GAGCAACUCCUGUACUGUA(dTdT) |
|  | Antisense(5‘-3‘) | UACAGUACAGGAGUUGCUC(dTdT) |

# Primers for proteins tested in the study

| β-ACTIN | Forward (5‘-3‘) | CATGTACGTTGCTATCCAGGC |
| --- | --- | --- |
|  | Reverse (5‘-3‘) | CTCCTTAATGTCACGCACGAT |
| AR | Forward (5‘-3‘) | CCAGGGACCATGTTTTGCC |
|  | Reverse (5‘-3‘) | CGAAGACGACAAGATGGACAA |
| KDM6B | Forward (5‘-3‘) | CACCCCAGCAAACCATATTATGC |
|  | Reverse (5‘-3‘) | CACACAGCCATGCAGGGATT |
| CCND1 | Forward (5‘-3‘) | GCTGCGAAGTGGAAACCATC |
|  | Reverse (5‘-3‘) | CCTCCTTCTGCACACATTTGAA |

# Primers of AR on KDM6B promotor in ChIP –PCR

| Site A | Forward (5‘-3‘) | TGATGAAGGCTTTAGGCAGAT |
| --- | --- | --- |
|  | Reverse (5‘-3‘) | AAGGCCCACGGAATAGTCTT |
| Site B | Forward (5‘-3‘) | CGTGTTGATCTCTGGAGCCTTT |
|  | Reverse (5‘-3‘) | CCTAGCCCTGTCCCCACTAGA |
| Site C | Forward (5‘-3‘) | TGAGGTGAGGGCCAACTTAG |
|  | Reverse (5‘-3‘) | CCCTTCCCAACCCTCATAGAC |

# Primers of Smad2/3 on CCND1 promotor in ChIP –PCR

| CCND1 ChIP-primer | Forward (5‘-3‘) | TGAGTCAGAATGGAGATCACTGT |
| --- | --- | --- |
|  | Reverse (5‘-3‘) | TCCCGTGCCGGCAATTT |

# Primers of H3K27me3 on CCND1 promotor in ChIP –PCR

| Site A | Forward (5‘-3‘) | CTGCCGGCCTTCCTAGTTG |
| --- | --- | --- |
|  | Reverse (5‘-3‘) | TGGGTCCCCCGGGATTTAG |
| Site B | Forward (5‘-3‘) | TAGGAACCTTCGGTGGTCTTG |
|  | Reverse (5‘-3‘) | CCGTGCCGGCAATTTAACC |
| Site C | Forward (5‘-3‘) | ATGAGCCCAGAATCCGCAGT |
|  | Reverse (5‘-3‘) | TTCAAGAAGCGCAGAGAAAGC |
