# Supplementary material for: KDM6B is an androgen regulated gene and plays oncogenic roles by demethylating H3K27me3 at cyclin D1 promoter in prostate cancer
Source: Cell Death Dis. 2021 Jan 6;12(1):2. doi: 10.1038/s41419-020-03354-4 (PMC7791132; doi:10.1038/s41419-020-03354-4)
Supplement: Supplementary file 8 — mass spectrometric analysis [file 41419_2020_3354_MOESM8_ESM.pdf]

Sample preparation for mass spectrometry analysis

The cells were washed with pre-cold PBS, collected and lysed in ST buffer (300mM Tris-HCl pH7.6, 2% SDS), incubated at 95^{0}C for 5 min. Cooled down and centrifuged at 14,000 rpm for 10 min. Pipetted the supernatant to a new tube and reduced with DTT. Mixed 200μg protein with 8M UA in the 10K filter unit and centrifuged at 14,000 g for 15 min. Added 200μl UA and centrifuged at 14,000 g for 15 min. Discarded the flow-through from the collection tube. Alkylated the proteins with IAA and incubated 45 min in the dark. Discarded the flow-through. Added 100μl UA and centrifuged at 14,000 g for 15 min and this step repeat once. Added 200μl 50mM ABC and centrifuged at 14,000 g for 15 min and this step repeat once. Changed a new collection tube and added 4μg trypsin and incubated at 37^{0}C for 16 h. Centrifuged at 14,000 g for 10 min and collected the flow-through to a new tube. Added 50mM ABC and centrifuged at 14,000 g for 10 min, collected the flow-through to the above tube and dried the sample with SpeedVac. The sample was dissolved in 0.1% TFA and desalted with C18 ZipTips and dried with SpeedVac, The sample were resuspened with 0.1% formic acid for mass spectrometry analysis.

Mass spectrometry analysis

The peptide samples were analyzed on Thermo Fisher LTQ Obitrap ETD mass spectrometry, Briefly, loaded sample onto an HPLC

chromatography system named Thermo Fisher Easy-nLC 1000 equipped with a C18 colume (1.8mm，0.15×1,00mm). Solvent A contained 0.1% formic acid and solvent B contained 100% acetonitrile. The elution gradient was from 4% to 18% in 182 min, 18% to 90% in 13 min solvent B at a flow rate of 300nL/min. Mass spectrometry analysis were carried out carried out at the AIMS Scientific Co.,Ltd.(Shanghai, China) in the positive-ion mode with an automated data-dependent MS/MS analysis with full scans (350-1600 m/z) acquired using FTMS at a mass resolution of 30,000 and the ten most intense precursor ions were selected for MS/MS. The MS/MS was acquired using higher-energy collision dissociation at 35% collision energy at a mass resolution of 15,000.

Database searching

Raw MS files were analyzed by MaxQuant (version 1.5.2.8), the parameter used for data analysis included trypsin as the protease with a maximum of two missed cleavages allowed. The mass tolerance for precursor ions and fragment ions was set to 20 ppm and 4.5ppm, respectively. The search included variable modifications of methionine oxidation and deamidation, and fixed modification of carbamidomethyl cysteine. Minimal peptide length was set to six amino acids and a maximum of two miscleavages was allowed. The false discovery rate (FDR) was set to 0.01 for peptide and protein identifications.

Relative protein quantification

In order to identify the interesting proteins, three technical repeats were carried out. The intensity for each protein were obtained from three technical repeats. The global analysis were carried by in-house program. The criteria for the interesting proteins was: the ratio of intensity between the experimental and control samples larger than 2 and p-value<0.05 was considered to be statistically significant.

Bioinformatic analysis

The Uniprot accession number of proteins were uploaded to the Database for Annotation, Visualization and Integrated Discovery (DAVID) bioinformatics resources (v6.8)(1). The cellular component, molecular function and biological process for the proteins were extracted and plotted with Origin. The KEGG pathways(2) were analyzed by the database KEGG: Kyoto Encyclopedia of Genes and Genomes (http://www.kegg.jp/) and plotted with Origin.

(1) Huang, D. W., Sherman, B. T., Tan, Q., Kir, J., Liu, D., Bryant, D., Guo, Y., Stephens, R., Baseler, M. W., Lane, H. C., and Lempicki, R. A. (2007) DAVID Bioinformatics Resources: expanded annotation database and novel algorithms to better extract biology from large gene lists. Nucleic Acids Res 35, W169-W175

(2) Kanehisa, M., Sato, Y., Kawashima, M., Furumichi, M., and Tanabe,

M. (2016) KEGG as a reference resource for gene and protein annotation. Nucleic Acids Res 44, D457-D462
