# Supplementary material for: KDM6B is an androgen regulated gene and plays oncogenic roles by demethylating H3K27me3 at cyclin D1 promoter in prostate cancer
Source: Cell Death Dis. 2021 Jan 6;12(1):2. doi: 10.1038/s41419-020-03354-4 (PMC7791132; doi:10.1038/s41419-020-03354-4)
Supplement: Supplementary file 9 — The details of RNA sequencing and data processing [file 41419_2020_3354_MOESM9_ESM.pdf]

仅供客户写英文文章时参考,分析内容和方法请以结题报告为准

——转录调控业务线

Method

Sample collection and preparation

- **RNA quantification and qualification**

RNA degradation and contamination was monitored on 1% agarose gels.

RNA purity was checked using the NanoPhotometer® spectrophotometer (IMPLEN, CA, USA) .

RNA concentration was measured using Qubit® RNA Assay Kit in Qubit®2.0 Flurometer (Life Technologies, CA, USA).

RNA integrity was assessed using the RNA Nano 6000 Assay Kit of the Bioanalyzer 2100 system (Agilent Technologies, CA, USA).

- **Library preparation for Transcriptome sequencing**

A total amount of 3 μg RNA per sample was used as input material for the RNA sample preparations. Sequencing libraries were generated using NEBNext® UltraTM RNA Library Prep Kit for Illumina® (NEB, USA) following manufacturer's recommendations and index codes were added to attribute sequences to each sample. Briefly, mRNA was purified from total RNA using poly-T oligo-attached magnetic beads. Fragmentation was carried out using divalent cations under elevated temperature in NEBNext First Strand Synthesis Reaction Buffer(5X). First strand cDNA was synthesized using random hexamer primer and M-MuLV

Reverse Transcriptase(RNase H^{-}). Second strand cDNA synthesis was subsequently performed using DNA Polymerase I and RNase H. Remaining overhangs were converted into blunt ends via exonuclease/polymerase activities. After adenylation of 3’ ends of DNA fragments, NEBNext Adaptor with hairpin loop structure were ligated to prepare for hybridization. In order to select cDNA fragments of preferentially 250~300 bp in length, the library fragments were purified with AMPure XP system (Beckman Coulter, Beverly, USA). Then 3 µl USER Enzyme (NEB, USA) was used with size-selected, adaptor-ligated cDNA at 37°C for 15 min followed by 5 min at 95 °C before PCR. Then PCR was performed with Phusion High-Fidelity DNA polymerase, Universal PCR primers and Index (X) Primer. At last, PCR products were purified (AMPure XP system) and library quality was assessed on the Agilent Bioanalyzer 2100 system.

- **Clustering and sequencing (Novogene Experimental Department)**

The clustering of the index-coded samples was performed on a cBot Cluster Generation System using TruSeq PE Cluster Kit v3-cBot-HS (Illumia) according to the manufacturer’s instructions. After cluster generation, the library preparations were sequenced on an Illumina Hiseq platform and 125 bp/150 bp paired-end reads were generated.

Data Analysis

- **Quality control**

Raw data (raw reads) of fastq format were firstly processed through in-house perl scripts. In this step, clean data (clean reads) were obtained by removing reads containing adapter, reads containing ploy-N and low quality reads from raw data. At the same time, Q20, Q30 and GC content the clean data were calculated. All the downstream analyses were based on the clean data with high quality.

Reads mapping to the reference genome

Reference genome and gene model annotation files were downloaded from genome website directly. Index of the reference genome was built using Hisat2 v2.0.5 and paired-end clean reads were aligned to the reference genome using Hisat2 v2.0.5. We selected Hisat2 as the mapping tool for that Hisat2 can generate a database of splice junctions based on the gene model annotation file and thus a better mapping result than other non-splice mapping tools.

Quantification of gene expression level

featureCounts v1.5.0-p3 was used to count the reads numbers mapped to each gene. And then FPKM of each gene was calculated based on the length of the gene and reads count mapped to this gene. FPKM, expected number of Fragments Per Kilobase of transcript sequence per Millions base pairs sequenced, considers the effect of sequencing depth and gene length for the reads count at the same time, and is currently the most commonly used method for estimating gene expression levels.

Differential expression analysis

(For DESeq2 with biological replicates) Differential expression analysis of two conditions/groups (two biological replicates per condition) was performed using the DESeq2 R package (1.16.1). DESeq2 provide statistical routines for determining differential expression in digital gene expression data using a model based on the negative binomial distribution. The resulting P-values were adjusted using the Benjamini and Hochberg's approach for controlling the false discovery rate . Genes with an adjusted P-value <0.05 found by DESeq2 were assigned as differentially expressed.

(For edgeR without biological replicates) Prior to differential gene expression analysis, for each sequenced library, the read counts were adjusted by edgeR program package through one scaling normalized factor. Differential expression analysis of two conditions was performed using the edgeR R package (3.18.1). The P values were adjusted using the Benjamini & Hochberg method. Corrected P-value of 0.05 and absolute foldchange of 2 were set as the threshold for significantly differential expression.

- **GO and KEGG enrichment analysis of differentially expressed genes**

Gene Ontology (GO) enrichment analysis of differentially expressed genes was implemented by the clusterProfiler R package, in which gene length bias wascorrected. GO terms with corrected Pvalue less than 0.05 were considered significantly enriched by differential expressed genes.

KEGG is a database resource for understanding high-level functions and utilities of the biological system, such as the cell, the organism and the ecosystem, from molecular-level information, especially large-scale molecular datasets generated by genome sequencing and other high-through put experimental technologies (http://www.genome.jp/kegg/). We used clusterProfiler R package to test the statistical enrichment of differential expression genes in KEGG pathways.

- **SNP analysis**

GATK2 (v3.7) software was used to perform SNP calling. Raw vcf files were filtered with GATK standard filter method and other parameters (cluster:3; WindowSize:35; QD < 2.0 o; FS > 30.0; DP < 10 and SnpEff software was used to annotation for the Variablesite.

- **AS analysis**

Alternative Splicing is an important mechanism for regulate the expression of genes and the variable of protein. rMATS(3.2.5) software was used to analysis the ASevent.

- **PPI analysis of differentially expressed genes**

PPI analysis of differentially expressed genes was based on the STRING database, which known and predicted Protein-Protein Interactions.

- **Fusion Analysis**

Fusion gene is refers to the two genes of all or part of the sequences perform fusion ,results of the chimeric gene, usually caused by reasons such as chromosome translocation and problem.We used star-fusion(1.2.0) software analysis and detection of fusion genes. Fusion gene list were filtered with star-fusion by standard filter method and other parameters(--annotate; --examine_coding_effect; --FusionInspector inspect; --denovo_reconstruct; --min_junction_reads 1 ; --min_sum_frags 2)

- References

- 1.Wang Z, Gerstein M, Snyder M. RNA-Seq: a revolutionary tool for transcriptomics[J]. Nature Reviews Genetics, 2009, 10(1): 57-63.
- 2.Parkhomchuk D, Borodina T, Amstislavskiy V, et al. Transcriptome analysis by strand-specific sequencing of complementary DNA[J]. Nucleic acids research, 2009, 37(18): e123-e123.
- 3.Andrews S. FastQC: A quality control tool for high throughput sequence data[J]. Reference Source, 2010.
- 4.Trapnell C, Pachter L, Salzberg S L. TopHat: discovering splice junctions with RNA-Seq[J]. Bioinformatics, 2009, 25(9): 1105-1111.(TopHat2)
- 5.Daehwan Kim, Ben Langmead, Steven L Salzberg.HISAT: a fast spliced aligner with low memory requirements. Nature methods,2015,12, 357–360.(HISAT)
- 6.Mortazavi A, Williams B A, McCue K, et al. Mapping and quantifying mammalian transcriptomes by RNA-Seq[J]. Nature methods, 2008, 5(7): 621-628.
- 7.Liao Y1, Smyth GK, Shi W. featureCounts: an efficient general purpose program for assigning sequence reads to genomicfeatures. Bioinformatics. 2014 ,30(7):923-30.(featureCounts)
- 8.Garber M, Grabherr M G, Guttman M, et al. Computational methods for transcriptome annotation and quantification using RNA-seq[J]. Nature methods, 2011, 8(6): 469-477.
- 9.Bray N, Pimentel H, Melsted P, et al. Near-optimal RNA-Seq quantification[J]. arXiv preprint arXiv:1505.02710, 2015.
- 10.Patro R, Mount S M, Kingsford C. Sailfish enables alignment-free isoform quantification from RNA-seq reads using lightweight algorithms[J]. Nature biotechnology, 2014, 32(5): 462-464.
- 11.Trapnell C, Roberts A, Goff L, et al. Differential gene and transcript expression analysis of RNA-seq experiments with TopHat and Cufflinks[J]. Nature protocols, 2012, 7(3): 562-578.(Tophat & Cufflinks)
- 12.Anders S, Huber W. Differential expression analysis for sequence count data[J]. Genome biol, 2010, 11(10): R106.
- 13.Love M I, Huber W, Anders S. Moderated estimation of fold change and dispersion for RNA-seq data with DESeq2[J]. Genome biology, 2014, 15(12): 1-21.(DESeq2)

- 14.Robinson M D, McCarthy D J, Smyth G K. edgeR: a Bioconductor package for differential expression analysis of digital gene expression data[J]. Bioinformatics, 2010, 26(1): 139-140.(edgeR)
- 15.Tarazona S, García-Alcalde F, Dopazo J, et al. Differential expression in RNA-seq: a matter of depth[J]. Genome research, 2011, 21(12): 2213-2223.(GOseq)
- 16.Young M D, Wakefield M J, Smyth G K, et al. Method Gene ontology analysis for RNA-seq: accounting for selection bias[J]. Genome Biol, 2010, 11: R14.
- 17.Kanehisa M, Goto S. KEGG: kyoto encyclopedia of genes and genomes[J]. Nucleic acids research, 2000, 28(1): 27-30.(KEGG)
- 18.Katz Y, Wang E T, Airoldi E M, et al. Analysis and design of RNA sequencing experiments for identifying isoform regulation[J]. Nature methods, 2010, 7(12): 1009-1015.
- 19.McKenna A, Hanna M, Banks E, et al. The Genome Analysis Toolkit: a MapReduce framework for analyzing next-generation DNA sequencing data[J]. Genome research, 2010, 20(9): 1297-1303.(GATK)
- 20.Jia W, Qiu K, He M, et al. SOAPfuse: an algorithm for identifying fusion transcripts from paired-end RNA-Seq data[J]. Genome Biol, 2013, 14(2): R12.(SOAPfuse)
- 21.Pastinen T. Genome-wide allele-specific analysis: insights into regulatory variation[J]. Nature Reviews Genetics, 2010, 11(8): 533-538.
- 22.Sun W. A statistical framework for eQTL mapping using RNA ‐ seq data[J]. Biometrics, 2012, 68(1): 1-111.
- 23.Giambartolomei C, Vukcevic D, Schadt E E, et al. Bayesian test for colocalisation between pairs of genetic association studies using summary statistics[J]. PLoS Genet, 2014, 10(5): e1004383.
- 24.Shen S., Park JW., Lu ZX., Lin L., Henry MD., Wu YN., Zhou Q., Xing Y. rMATS: Robust and Flexible Detection of Differential Alternative Splicing from Replicate RNA-Seq Data.(rMATS).
- 25.Jiao Y, Widschwendter M, Teschendorff A E. A systems-level integrative framework for genome-wide DNA methylation and gene expression data identifies differential gene expression modules under epigenetic control[J]. Bioinformatics, 2014, 30(16): 2360-2366.
- 26.Lee H, Yang Y, Chae H, et al. BioVLAB-MMIA: a cloud environment for microRNA and mRNA integrated analysis (MMIA) on Amazon EC2[J]. NanoBioscience, IEEE Transactions on, 2012, 11(3): 266-272.
- 27.Conesa A, Madrigal P, Tarazona S, et al. A Survey of Best Practices for RNA-seq Data Analysis[J]. 2016.
- 28.View ORCID ProfileBrian Haas, Alexander Dobin, Nicolas Stransky,et al. STAR-Fusion: Fast and Accurate Fusion Transcript Detection from RNA-Seq[J]. biorxiv, 2017.(STAR-Fusion)
  29.Janet Piñero Àlex Bravo Núria Queralt-Rosinach, et al. DisGeNET: a comprehensive platform integrating information on human disease-associated genes and variants [J]. Nucleic Acids Research, 2017, 45(D1): D833-D839.
