# Supplementary material for: KDM6B is an androgen regulated gene and plays oncogenic roles by demethylating H3K27me3 at cyclin D1 promoter in prostate cancer
Source: Cell Death Dis. 2021 Jan 6;12(1):2. doi: 10.1038/s41419-020-03354-4 (PMC7791132; doi:10.1038/s41419-020-03354-4)
Supplement: Supplementary file 10 — Dual-luciferase reporter assay report [file 41419_2020_3354_MOESM10_ESM.pdf]

合同号：HY70460-C

双萤光素酶报告基因检测（启动子活性检测）实验

和元生物技术（上海）股份有限公司，2020 年 01 月 04 日

摘要：通过双萤光报告基因系统检测转录因子 AR 对启动子 KDM6B 活性的影响，结合定点突变技术可以进一步确定转录因子 AR 与启动子 KDM6B 的作用位点。

关键词：启动子，转录因子，Dual-Reporter Assay

OBiO® 和元上海

整合你我资源 服务生命科学

一、实验原理

蛍光素酶是理想的报告基因，因为哺乳动物细胞中不含内源性萤光素酶，一旦转录完成立刻就生成功能性的萤光素酶。单报告基因实验往往会受到各种实验条件的影响，而双报告基因则通过共转染的“对照”作为内参为试验提供一基准线，从而可以在最大程度上减小细胞活性和转染效率等外在因素对实验的影响，使得数据结果更为可信。Dual-Luciferase®双萤光素酶报告基因检测系统在细胞中同时表达萤火虫萤光素酶和海肾萤光素酶，两者没有种源同源性并对应不同的反应底物，故而没有交叉干扰。得益于超强的光信号和超高的信噪比。

转录因子是一种具有特殊结构、行使调控基因表达功能的蛋白质分子，也称为反式作用因子。某些转录因子仅与其靶启动子中的特异序列结合，这些特异性的序列被称为顺式因子，转录因子的 DNA 结合域和顺式因子实现共价结合，从而对基因的表达起抑制或增强的作用。萤光素酶报告基因实验（luciferase Assay）是检测这类转录因子和其靶启动子中的特异顺序结合的重要手段，其原理简述如下：(1)用靶启动子的特定片段替换报告基因质粒(如 pGL4.10-basic)的启动子区。(2)将要检测的转录因子表达质粒与报告基因质粒共转。如果该转录因子能够激活靶启动子，则萤光素酶就会表达，且其表达量与转录因子的作用强度成正比。(3)加入特定的底物，萤光素酶与底物反应，产生萤光素，检测荧光的强度可以对萤光素酶的活性定量，进而判断该转录因子能否与靶启动子片段发生作用（及其作用强度）。

二、实验目的

通过双萤光报告基因系统检测转录因子 AR 对启动子 KDM6B 活性的影响，

结合定点突变技术可以进一步确定转录因子 AR 与启动子 KDM6B 的作用位点。

三、实验材料

1. 质粒与细胞株:

实验质粒 pGL4.10（H352，和元构建）；

pLenti-EF1a-EGFP-P2A-Puro-CMV-MCS-3Flag（H149，和元构建）；

pLenti-EF1a-EGFP-P2A-Puro-CMV-AR-3Flag（H8039，和元构建）；

pGL4.10-KDM6B promoter（WT）（H14957，和元构建）；pGL4.10- KDM6B promoter（MUT）（H14958，和元构建）； pRL-CMV（H321，Promega）；293T 细胞株来源于库存，37℃，5% CO_2。

2. 实验试剂:

lipofectamine 2000 （ 11668-019, invitrogen ）；胎 牛 血 清 (FBS)（SV30087.02，Hyclone）； DMEM（12800-017，GIBCO）；胰酶（Trypsin-EDTA）（11668-500，Invitrogen）；96 孔板（3524，Corning）；Dual-Luciferase Reporter Assay System（E1910， Promega）；Opti-MEM（31985-062, invitrogen）。

3. 仪器设备:

荧光显微镜（DMi8，LEICA）；超净台（SW-CJ-1F，苏州净化）；CO2 培养箱（311，Thermo）；低速离心机（TDZ4-WS，上海湘仪）；血球计数板 Bright Line counting chamber (1492，Hausser Scientific)；水浴锅（HWS12，上海一恒）；酶标仪（Spark 10M，TECAN）。

OBiO® 和元上海
整合你我资源 服务生命科学

四、实验步骤

1. 质粒转染细胞：

1) 细胞铺板：将细胞按 70%的汇合度接种到 96 孔板。

2) 转染：24 小时后转染萤光素酶报告基因质粒和反式作用因子基因质粒，每个样品设置 6 个复孔。

a) 配制 DNA 和转染试剂: 96 孔板转染质粒时每孔比例为萤火虫萤光素酶载体（Firefly）:海肾萤光素酶载体（Renilla）:转染试剂=0.1μg：0.005 μg：0.25μL。

b) 配制转录因子和转染试剂：96 孔板转染质粒时每孔比例为（转录因子质粒、对照质粒和客供质粒）: 转染试剂=0.1ug：0.25uL。

c) 稀释好 DNA 和转录因子及转染试剂，常温孵育 5min。

d) 将稀释好的 DNA 和转录因子载体分别和转染试剂混匀，常温孵育 20min。

e) 每孔弃去 50uL 培养基，将 50μL 质粒转染混合液添加到每孔细胞样品中。

f) 转染 6 小时后，换新鲜完全培养基。

2. 双报告基因检测：

a) 质粒共转染 48 小时后，弃去培养基，用 100μL 1XPBS 洗 1 遍。

b) 倾斜 96 孔板，吸干剩余的 PBS。

c) 去离子水将 5XPLB(Passive Lysis Buffer)稀释成 1XPLB，使用前放到常温。

d) 每孔加 100μL 稀释好的 1X PLB，摇床常温条件下摇 15min，进行裂解。

OBiO® 和元上海 整合你我资源 服务生命科学

e) 白色不透光的 96 孔酶标板中每孔加步骤 4 的上清液 5μL，加入 50μL 预先混好的 LAR II，2s 后测数据。

f) 每孔添加 50μL 预先混好的 Stop&Glo Reagent，静止 2s 后，测数据。

注意：进行 Luciferase 活性检测时，需在避光条件下进行。

3. 统计学分析：

GraphPad Prism（Ver. 5，GraphPad Software） 作图，并进行双侧 t 检验。

五、实验结果

表 1. 空白校准后的数据

Data		Group 1	Group 2	Group 3	Group 4	Group 5	Group 6
Firefly luciferase	H352+H149	4266	4855	2420	2835	3877	4769
	H352+H8039	5074	3865	4208	3658	4324	4857
	H14957+H149	323803	335926	321718	243000	328166	351168
	H14957+H8039	276695	302124	238473	275409	277526	271021
	H14958+H149	269159	266766	237816	233829	305785	262390
	H14958+H8039	296319	299716	297822	238062	292664	270033
Renilla luciferase	H352+H149	97623	113804	59187	62910	91000	110854
	H352+H8039	175814	149056	155334	129629	155445	149558
	H14957+H149	101898	95721	94254	77331	93302	106406
	H14957+H8039	162167	183203	142050	155069	157731	150767
	H14958+H149	117787	126165	112340	121302	141849	110896
	H14958+H8039	199807	211190	188572	165209	197486	172916
Ratio	H352+H149	0.0437	0.0427	0.0409	0.0451	0.0426	0.0430
	H352+H8039	0.0289	0.0259	0.0271	0.0282	0.0278	0.0325
	H14957+H149	3.1777	3.5094	3.4133	3.1423	3.5172	3.3003
	H14957+H8039	1.7062	1.6491	1.6788	1.7760	1.7595	1.7976
	H14958+H149	2.2851	2.1144	2.1169	1.9277	2.1557	2.3661

	H14958+H8039	1.4830	1.4192	1.5794	1.4410	1.4819	1.5616

Firefly luciferase的数据除以Renialla luciferase的数据 ,用平均值及归一化后平均值做图。

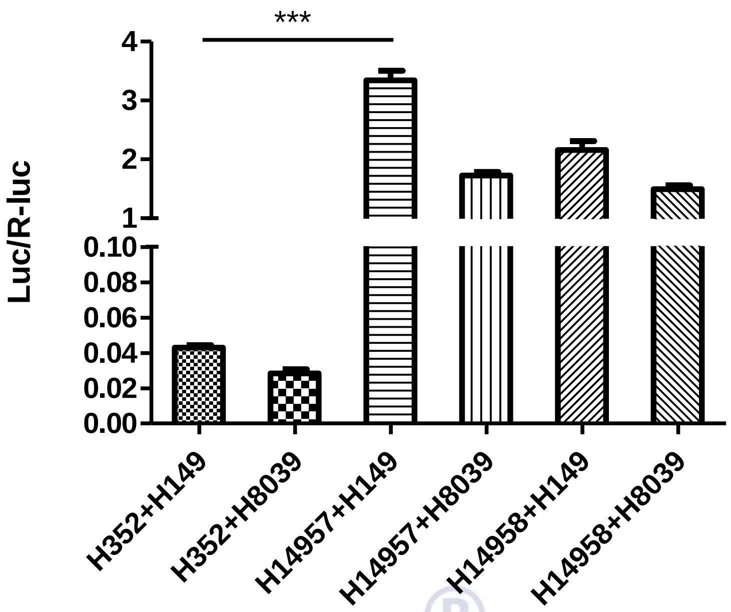

图 1. 检测转录因子 AR 对 KDM6B 启动子活性的影响

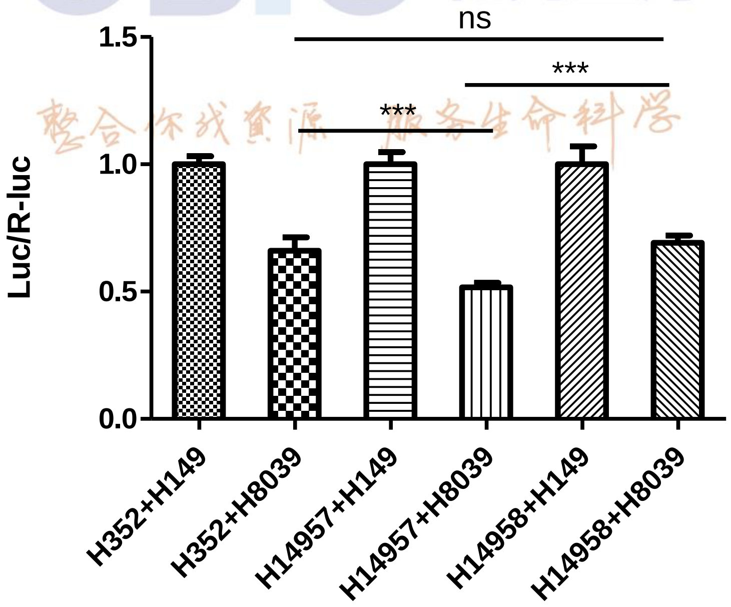

图 2. 检测转录因子 AR 对 KDM6B 启动子活性的影响

pGL4.10 H352

pLenti-EF1a-EGFP-P2A-Puro-CMV-MCS-3Flag H149

pLenti-EF1a-EGFP-P2A-Puro-CMV-AR-3Flag H8039

pGL4.10-KDM6B promoter（WT） H14987

pGL4.10- KDM6B promoter（MUT） H14958

ns 表示无差异, *表示 p<0.05, **表示 p<0.01,***表示 p<0.001

结论：

由图 1 可以看出，启动子活性为空载活性的 77.77 倍。

由图 2 可以看出 转录因子 AR 对空载体活性有明显的抑制作用，下降了 33.94%。

和空载+转录因子组相比，KDM6B 野生型启动子+转录因子组活性明显降低，降低了 14.38%（p<0.0001）; 而在结合位点突变后，这种激活作用明显减弱（p<0.0001），回升了 17.47%。同时，和空载+转录因子组相比，KDM6B 突变型启动子+转录因子组活性无明显变化（p=0.2347）。由此可以说明 AR 通过该结合位点调控 KDM6B 启动子的活性。

® OBiO 和元上海

六、参考文献：

1. Yang HM, Do HJ, Kim DK, Park JK, Chang WK, Chung HM, Choi SY, Kim JH.Transcriptional regulation of human Oct-4 by steroidogenic factor-1.J Cell Biochem. 2007;101(5):1198-1209.
2. Lorenz WW, McCann RO, Longiaru M, Cormier MJ.Isolation and expression of a cDNA encoding Renilla reniformis luciferase.Proc Natl Acad Sci U S A. 1991 May 15;88(10):4438-42.
3. Farr A, Roman A. pitfall of using a second plasmid to determine transfection efficiency.Nucleic Acids Res. 1992 Feb 25;20(4):920.
4. Sherf, B.A. et al. Dual-Luciferase® reporter assay: An advanced co-reporter technology integrating firefly and Renilla luciferase assays. 1996 Promega Notes 57, 2-9.
