# Supplementary material for: KDM6B is an androgen regulated gene and plays oncogenic roles by demethylating H3K27me3 at cyclin D1 promoter in prostate cancer
Source: Cell Death Dis. 2021 Jan 6;12(1):2. doi: 10.1038/s41419-020-03354-4 (PMC7791132; doi:10.1038/s41419-020-03354-4)
Supplement: Supplementary file 11 — C4-2B authentication [file 41419_2020_3354_MOESM11_ESM.pdf]

上海艾博思生物科技有限公司

Shanghai Integrated Biotech Solutions Co.,Ltd

地址：上海浦东张江高科技园区紫薇路 750 弄 10 号 202 室

邮编：201203　电话：021-3877863　传真：021-38770863

Cell Line Authentication Report

Customer: Wang Chao

Institution: Changhai Hospital

Quotation Number: 80-118452019

Completion Date：12/29/2017

1. **Sample ID: C4-2B**
2. **Original Material: Cell pellets**
3. **Methods:**

1).Genomic DNA was extracted from the cell pellets provided by the customer.

2).Samples, together with positive and negative control were amplified using GenePrint 10 System (Promega).

3). Amplified products were processed using the ABI3730xl Genetic Analyzer.

4).Data were analyzed using GeneMapper4.0 software and then compared with the ATCC, DSMZ or JCRB databases for reference matching.

4. **Results:**

1) 10 Loci STR Profile：

Genetic Site	Customer sample			
(Locus)	C4-2B			
Amelogenin	X		Y	
CSF1PO	10		11	
D13S317	10		11	
D16S539	11			
D5S818	11		12	
D7S820	8	9	11	12
THO1	9			
TPOX	8		9	
vWA	16		17	18
D21S11	29		32.2	

[键入文字]

上海艾博思生物科技有限公司

Shanghai Integrated Biotech Solutions Co.,Ltd

地址：上海浦东张江高科技园区紫薇路 750 弄 10 号 202 室

邮编：201203　　电话：021-3877863　传真：021-38770863

<<<If the Percent match is not 100%, search for reference matchingwith the ATCC, DSMZ or JCRB databases and add the match results.

Addendum: Comparative output from the ATCC STR Profile database

Result of STR matching analysis by your data.

- DSMZ Profile Database -

A graphical presentation is shown at the bottom of this page.

EV	Cell No.	Cell name	Locus names									Figures
			D5S818	D13S317	D7S820	D16S539	VWA	TH01	AM	TPOX	CSF1PO	
	Query (Your Cell)		*11,12*	*10,11*	*8,9,11,12*	*11,11*	*16,17,18*	*9,9*	*X,Y*	*8,9*	*10,11*	
0.85(34/40)	RCB2144	LNCap.FGC	11,12	10,12	9,9	11,11	16,17,18	9,9	X,Y	8,9	10,11	-
0.82(32/39)	RCB2266	HE50	11,12	10,12	8,9	11,11	17,17	9,9	X,Y	8,9	10,11	-
0.77(30/39)	256	LNCAP	11,12	10,12	9.1,10.3	11,11	16,18	9,9	X,Y	8,9	10,11	-
0.77(30/39)	CRL-1740	LNCaP clone FGC	11,12	10,12	9.1,10.3	11,11	16,18	9,9	X,Y	8,9	10,11	-
0.73(30/41)	HTB-68	SK-MEL-2	11,12,13	10,11,13	11,12	8,9	16,17	9,9	X,Y	8,9	10,12	-
0.72(28/39)	CRL-2220	CA-HPV-10	11,12	11,12	10,11	11,11	16,17	6,8	X,Y	8,9	10,11	-
0.72(28/39)	JCRB0041	HLCL-1	11,13	10,12	8,12	11,11	16,17	9,9	X,Y	9,11	11,12	-
0.71(30/42)	CRL-5833	NCI-H630	12,12	11,12,13	8,11,12	11,12	15,16,17	7,9	X,Y	8,9	10,11	-
0.67(26/39)	CRL-2691	CCD-1135Sk	12,13	8,11	8,11	9,12	16,18	9,9	X,Y	8,9	10,13	-
0.67(26/39)	JCRB1217	MT-3	11,11	10,11	10.3,12	9,11	14,17	9,9	X,Y	8,9	10,12	-
0.67(26/39)	JCRB3010	FA20P	10,12	7,11	11,12	11,11	16,17	9,10	X,Y	8,9	12,13	-
0.67(26/39)	RCB0197	HUC-Fm	11,14	11,12	8,12	10,11	16,18	9,9	X,Y	8,11	9,11	-
0.67(26/39)	RCB0492	OTCD1TKB	12,13	11,12	11,12	10,11	16,17	9,9	X,Y	8,11	11,12	-

>>>

上海艾博思生物科技有限公司

Shanghai Integrated Biotech Solutions Co.,Ltd

地址：上海浦东张江高科技园区紫薇路 750 弄 10 号 202 室

邮编：201203　电话：021-3877863　传真：021-38770863

2) **Electrophoretogram**

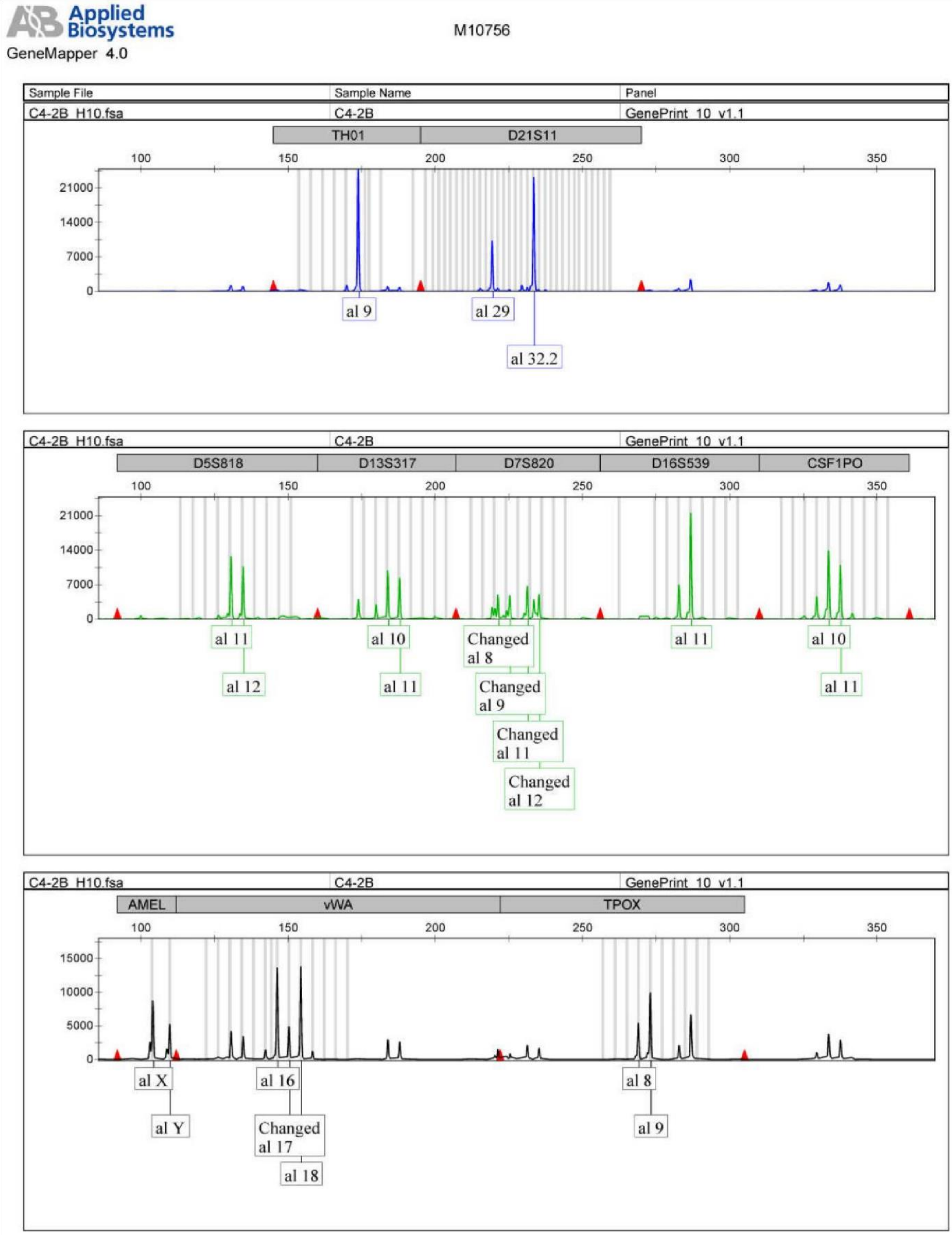

Note：Raw data in appendix
