# Supplementary material for: KDM6B is an androgen regulated gene and plays oncogenic roles by demethylating H3K27me3 at cyclin D1 promoter in prostate cancer
Source: Cell Death Dis. 2021 Jan 6;12(1):2. doi: 10.1038/s41419-020-03354-4 (PMC7791132; doi:10.1038/s41419-020-03354-4)
Supplement: Supplementary file 12 — Lncap authentication [file 41419_2020_3354_MOESM12_ESM.pdf]

# Cell Line Authentication Service

## STR Profiling Report

**Sample From:** Shanghai Changhai Hospital

**Sample Type:** Cell Line

**Testing Method:** STR Genotyping

**Report Time:** 4/19/2019


**Add:** Room 205,.885 Wangjiashe Rd,Songjiang Dist,Shanghai/ Tel:+86-021-33559491
Homepage: http://www.biowing.com.cn/ E-mail: market@biowing.com.cn

# COMPANY STATEMENT

1. THIS REPORT IS ONLY RESPONSIBLE FOR THE SAMPLES ANALYZED.
2. THE TESTING RESULTS AND THE ORGANIZATION NAME WILL NOT BE USED FOR ADVERTISEMENT, COMMERCIAL EXHIBITIONS, COMMERCIAL PERFORMANCE AND OTHER COMMERCIAL ACTIVITIES.
3. OBJECTIONS SHOULD BE RAISED WITHIN FIFTEEN DAYS AFTER THE RECEIPT OF THIS REPORT.
4. THE PAPER REPORT WITH CONTENT ALTERING, ADDING OR WITHOUT THE STAMPED SEAL OF THE COMPANY ARE INVALID.

**Testing Company:** Shanghai Biowing Applied Biotechnology Co. Ltd
**Address:** Room 205, NO.885 Wangjiashe Road, Songjiang District, Shanghai
**Tel:** +86-021-33559491
**Contact:** Wenyao Zhang
**E-mail:** market@biowing.com.cn

# Cell Line Authentication – STR Profiling Report

## Sample code

Table 1. Sample Code

| Customer's code | Company Code |
|---|---|
| 1 | 20190411-01 |

**Sample Number** : 1

**Sample Type**: Cell line

**Testing Type**: STR

**Testing Method**:

DNA was extracted by a commercial kit from CORNING (AP-EMN-BL-GDNA-250G). The twenty STRs including Amelogenin locus were amplified by six multiplex PCR and separated on ABI 3730XL Genetic Analyzer. The signals were then analyzed by the software GeneMapper.

## Data Interpretation:

Cell lines were authenticated using Short Tandem Repeat (STR) analysis asdescribed in 2012 in ANSI Standard (ASN-0002) by the ATCC Standards Development Organization (SDO) and in Capes-Davis et al., Match criteria for human cell line authentication: Where do we draw the line? Int J Cancer.2013;132(11):2510-9.


Add: Room 205,.885 Wangjiashe Rd,Songjiang Dist,Shanghai/ Tel:+86-021-33559491
Homepage: http://www.biowing.com.cn/ E-mail: market@biowing.com.cn

# Test Results

## 1. STR profile

Table 2. STR and Amelogenin Genotyping Results of Cell line.

| Loci | Sample information | | | Cell Bank information | | |
|---|---|---|---|---|---|---|
| | Sample name : 1 | | | Cell line name : LNCAP | | |
| | **Allele1** | **Allele2** | **Allele3** | **Allele1** | **Allele2** | **Allele3** |
| D5S818 | 11 | 12 | | 11 | 12 | |
| D13S317 | 10 | 12 | | 10 | 12 | |
| D7S820 | 9.1 | 10.3 | | 9.1 | 10.3 | |
| D16S539 | 11 | 11 | | 11 | 11 | |
| VWA | 16 | 18 | | 16 | 18 | |
| TH01 | 8 | 9 | | 9 | 9 | |
| AMEL | X | Y | | X | Y | |
| TPOX | 8 | 9 | | 8 | 9 | |
| CSF1PO | 10 | 11 | | 10 | 11 | |
| D12S391 | 20 | 21 | 22 | | | |
| FGA | 19 | 20 | | | | |
| D2S1338 | 16 | 16 | | | | |
| D21S11 | 29 | 32.2 | | | | |
| D18S51 | 11 | 12 | | | | |
| D8S1179 | 12 | 14 | | | | |
| D3S1358 | 16 | 16 | | | | |
| D6S1043 | 11 | 18 | | | | |
| PENTAE | 12 | 16 | | | | |
| D19S433 | 13.2 | 15 | 17 | | | |
| PENTAD | 12 | 12.3 | | | | |
| D1S1656 | 15 | 16 | | | | |


**Add:** Room 205,.885 Wangjiashe Rd,Songjiang Dist,Shanghai/ Tel:+86-021-33559491
Homepage: http://www.biowing.com.cn/ E-mail: market@biowing.com.cn

## 2. database annotation

Figure 1. STR matching analysis

| EV | Cell No. | Cell name | Locus names | | | | | | | | |
|---|---|---|---|---|---|---|---|---|---|---|---|
| | | | D5S818 | D13S317 | D7S820 | D16S539 | VWA | TH01 | AM | TPOX | CSF1PO |
| | *Query (Your Cell)* | | *11,12* | *10,12* | *9.1,10.3* | *11,11* | *16,18* | *8,9* | *X,Y* | *8,9* | *10,11* |
| 0.94(34/36) | 256 | LNCAP | 11,12 | 10,12 | 9.1,10.3 | 11,11 | 16,18 | 9,9 | X,Y | 8,9 | 10,11 |

**Note:** The STR online match analysis of the test cell against DSMZ database, showing cell number (Cell No.) and cell name.

## 3. Authentication

☐ The submitted sample profile is human, but not a match for any profile in the DSMZ.

☑ The submitted profile is an basic match for the following human cell line(s) in the DSMZ STR database (8 core loci plus Amelogenin): **LNCAP**.

☐ The submitted profile is similar to the following DSMZ human cell line: /.

- **Note:** A cell line can be considered to be authenticated when 80% (exact match) of the alleles in its STR profile match profiles from tissue or other cell line samples from that donor or from database. Cell lines with between a 55% to 80% (similar) match require further profiling for investigation of relatedness.



Add: Room 205,.885 Wangjiashe Rd,Songjiang Dist,Shanghai/ Tel:+86-021-33559491
Homepage: http://www.biowing.com.cn/ E-mail: market@biowing.com.cn

# Appendix

1. Genotyping Strategy and Site Distribution

Table S1. Experimental Strategy and Sites

| | Strategy 1 | Strategy 2 | Strategy 3 | Strategy 4 |
|---|---|---|---|---|
| 1 | D3S1358 | D8S1179 | D19S433 | AMEL |
| 2 | VWA | D21S11 | TH01 | D1S1656 |
| 3 | D7S820 | D16S539 | D13S317 | D5S818 |
| 4 | CSF1PO | D2S1338 | TPOX | D12S391 |
| 5 | PENTAE | PENTAD | D18S51 | FGA |
| 6 | | | D6S1043 | |

*The allele match algorithm compares the 8 core loci plus amelogenin only, even though alleles from all lociwill be reported when available.*

2. DSMZ tools was used to carry on the cell line comparison, which contains 2455 cell lines STR data from ATCC, DSMZ, JCRB ,ECACC， GNE and RIKEN databases. If the cell is not included in the above cell library, users need to compared with other databases.

**Technician:** Jianan Zhang

**Checked by:** Ning Qian

**Issued by**: Yang Bai

**Issue date:** 4/19/2019


**Add:** Room 205,.885 Wangjiashe Rd,Songjiang Dist,Shanghai/ Tel:+86-021-33559491
Homepage: http://www.biowing.com.cn/ E-mail: market@biowing.com.cn
