# Supplementary material for: KDM6B is an androgen regulated gene and plays oncogenic roles by demethylating H3K27me3 at cyclin D1 promoter in prostate cancer
Source: Cell Death Dis. 2021 Jan 6;12(1):2. doi: 10.1038/s41419-020-03354-4 (PMC7791132; doi:10.1038/s41419-020-03354-4)

SHANGHAI BIOWING BIOTECHNOLOGY Co. LTD
*Your Intimate Partner in Bioscience!*

# Cell Line Authentication Service

## STR Profiling Report

Sample Type: Cell Line

Sample from: Shanghai Changhai Hospital

Testing Method: STR Genotyping

Report Time: June 8, 2018

# COMPANY STATEMENT

1. THIS REPORT IS ONLY RESPONSIBLE FOR THE SAMPLES ANALYZED.
2. THE TESTING RESULTS AND THE ORGANIZATION NAME WILL NOT BE USED FOR ADVERTISEMENT, COMMERCIAL EXHIBITIONS, COMMERCIAL PERFORMANCE AND OTHER COMMERCIAL ACTIVITIES.
3. OBJECTIONS SHOULD BE RAISED WITHIN FIFTEEN DAYS AFTER THE RECEIPT OF THIS REPORT.
4. THE PAPER REPORT WITH CONTENT ALTERING, ADDING OR WITHOUT THE STAMPED SEAL OF THE COMPANY ARE INVALID.

**Testing Company:** Shanghai Biowing Applied Biotechnology Co. Ltd

**Address:** Room 4F, 8 th Buiding,Guiguo Garden,NO.471 Guiping Road,Caohejing Development Zone,Shanghai

**Tel:** +86-021-33559491

**Contact:** YiQun Chen

**E-mail:** biowing@vip.163.com

# Cell Line Authentication – STR Profiling Report

## Sample code

Table 1. Sample Code

| Customer's code | Company Code |
|---|---|
| PC-3 | 20180601-03 |

## Sample Number:1

## Sample Type: Cell line

## Testing Type: STR

## Sample From: Shanghai Changhai Hospital

## Testing Method:

DNA was extracted by a commercial kit from CORNING (AP-EMN-BL-GDNA-250G). The twenty STRs including Amelogenin locus were amplified by six multiplex PCR and separated on ABI 3730XL Genetic Analyzer. The signals were then analyzed by the software GeneMapper.

## Data Interpretation:

Cell lines were authenticated using Short Tandem Repeat (STR) analysis as described in 2012 in ANSI Standard (ASN-0002) by the ATCC Standards Development Organization (SDO) and in Capes-Davis et al., Match criteria for human cell line authentication: Where do we draw the line? Int J Cancer.2013;132(11):2510-9.

## Test Results:

### 1. Result

Table 2. Matching information on the cell lines

| Sample Code | Multi-allele | Cell line matched | Cell Bank | EV | Percentage |
|---|---|---|---|---|---|
| 20180601-03 | No | PC-3 | DSMZ | 1.00 | 9/9 |

- Multi-allele means some STR contain more than two loci.

## 2. Sample Description

20180601-03:

A. The STR results showed that no multiple alleles were found in this cell line, and no cross contamination was found in the cell line.

B. The DNA of the cell lines found to perfect match the type of cell lines in a cell line retrieval, DSMZ database shows that cells called **PC-3.** corresponding to the cell number **465.**

| EV | Cell No. | Cell name | D5S818 | D13S317 | D7S820 | D16S539 | VWA | TH01 | AM | TPOX | CSF1PO |
|---|---|---|---|---|---|---|---|---|---|---|---|
| | Query (Your Cell) | | 13,13 | 11,11 | 8,11 | 11,11 | 17,17 | 6,7 | X,X | 8,9 | 11,11 |
| 1.00(36/36) | 465 | PC-3 | 13,13 | 11,11 | 8,11 | 11,11 | 17,17 | 6,7 | X,X | 8,9 | 11,11 |
| 1.00(36/36) | CRL-1435 | PC-3 | 13,13 | 11,11 | 8,11 | 11,11 | 17,17 | 6,7 | X,X | 8,9 | 11,11 |

Locus names: D5S818 – CSF1PO

## 3. Genotyping Result

Table 3. STR and Amelogenin Genotyping Results of Cell line 20180601-03

| Loci | Sample information | | | Cell Bank information | | |
|---|---|---|---|---|---|---|
| | Sample name：PC-3 | | | Cell line name：PC-3 | | |
| | **Allele1** | **Allele2** | **Allele3** | **Allele1** | **Allele2** | **Allele3** |
| D5S818 | 13 | 13 | | 13 | 13 | |
| D13S317 | 11 | 11 | | 11 | 11 | |
| D7S820 | 8 | 11 | | 8 | 11 | |
| D16S539 | 11 | 11 | | 11 | 11 | |
| VWA | 17 | 17 | | 17 | 17 | |
| TH01 | 6 | 7 | | 6 | 7 | |
| AMEL | X | X | | X | X | |
| TPOX | 8 | 9 | | 8 | 9 | |
| CSF1PO | 11 | 11 | | 11 | 11 | |
| D12S391 | 21 | 21 | | | | |
| FGA | 24 | 24 | | | | |
| D2S1338 | 18 | 20 | | | | |
| D21S11 | 29 | 31.2 | | | | |
| D18S51 | 14 | 15 | | | | |

| | | | |
|---|---|---|---|
| D8S1179 | 13 | 13 | |
| D3S1358 | 16 | 16 | |
| D6S1043 | 18 | 18 | |
| PENTAE | 10 | 17 | |
| D19S433 | 14 | 14 | |
| PENTAD | 9 | 9 | |

Others:

1. Genotyping Strategy and Site Distribution

Attached Table. Experimental Strategy and Sites

| | Strategy 1 | Strategy 2 | Strategy 3 | Strategy 4 |
|---|---|---|---|---|
| 1 | TH01 | TPOX | D3S1358 | AMEL |
| 2 | D12S391 | VWA | D13S317 | D5S818 |
| 3 | D7S820 | D8S1179 | D6S1043 | D2S1338 |
| 4 | CSF1PO | PENTAD | D16S539 | D21S11 |
| 5 | FGA | | D19S433 | D18S51 |
| 6 | PENTAE | | | |

*The allele match algorithm compares the 8 core loci plus amelogenin only, even though alleles from all loci will be reported when available.*

2. DSMZ tools was used to carry on the cell line comparison, which contains 2455 cell lines STR data from ATCC, DSMZ, JCRB ,ECACC，GNE and RIKEN databases. If the cell is not included in the above cell library, users need to compared with other databases.

Technician: Menglu Shen
Check: Yang Bai
Person in Charge: Yiqun Chen

Issue date: June 8, 2018

| Sample File | Sample Name | Panel | SQO | OS | SQ |
|---|---|---|---|---|---|
| 39_G05_CellLineAthentication-1-0606.fsa | PC-3 | 21Plex_STR_Panel_v0.4 | | | |

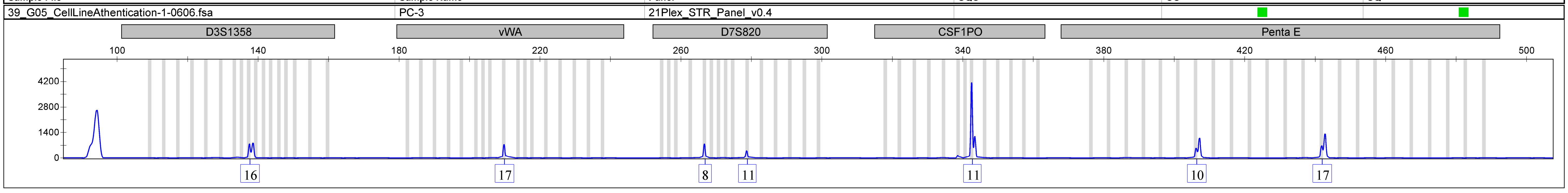


| Sample File | Sample Name | Panel | SQO | OS | SQ |
|---|---|---|---|---|---|
| 39_G05_CellLineAthentication-1-0606.fsa | PC-3 | 21Plex_STR_Panel_v0.4 | | | |

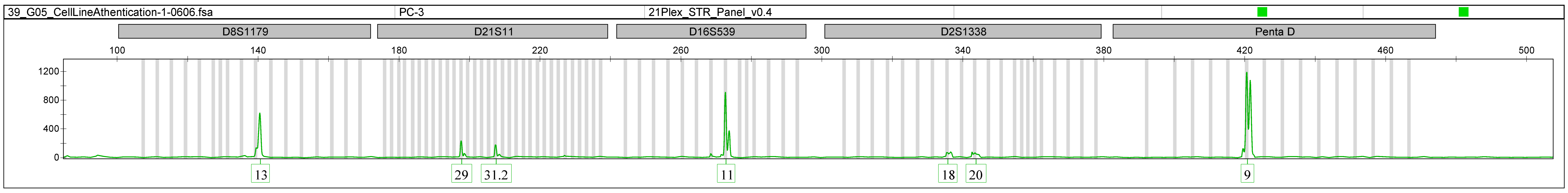


| Sample File | Sample Name | Panel | SQO | OS | SQ |
|---|---|---|---|---|---|
| 39_G05_CellLineAthentication-1-0606.fsa | PC-3 | 21Plex_STR_Panel_v0.4 | | | |

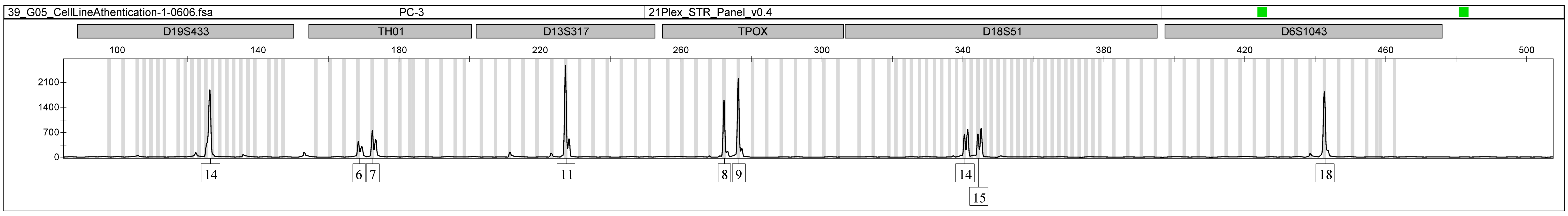


| Sample File | Sample Name | Panel | SQO | OS | SQ |
|---|---|---|---|---|---|
| 39_G05_CellLineAthentication-1-0606.fsa | PC-3 | 21Plex_STR_Panel_v0.4 | | | |

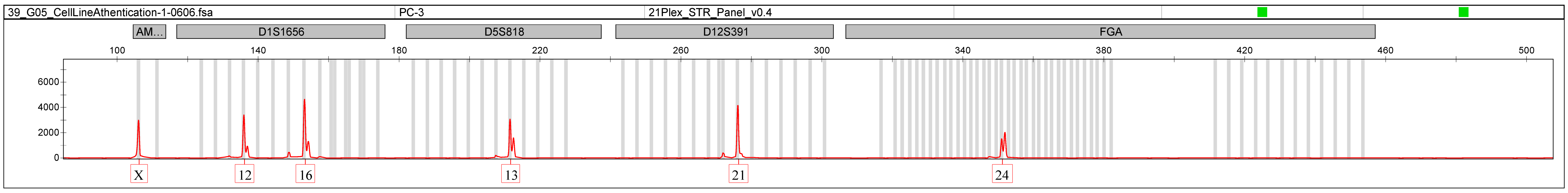

Supplement: Supplementary file 13 — PC-3 authentication [file 41419_2020_3354_MOESM13_ESM.pdf]
